# Quantitative Measurement of Thermal Conductivity by SThM Technique: Measurements, Calibration Protocols and Uncertainty Evaluation

**DOI:** 10.3390/nano13172424

**Published:** 2023-08-25

**Authors:** Nolwenn Fleurence, Séverine Demeyer, Alexandre Allard, Sarah Douri, Bruno Hay

**Affiliations:** 1 Laboratoire National de Métrologie et d’Essais (LNE), 29, Avenue Roger Hennequin, 78197 Trappes, France; severine.demeyer@lne.fr (S.D.); sarah.douri@lne.fr (S.D.); bruno.hay@lne.fr (B.H.); 2 Detection, Sensors and Measurements Laboratory, Ifremer, 1625 Route de Sainte-Anne, 29280 Plouzané, France; 3 CETHIL UMR5008, CNRS, INSA-Lyon, Université Claude Bernard Lyon 1, 69621 Villeurbanne, France

**Keywords:** uncertainty, thermal conductivity, SThM, nanoscale, Monte Carlo method, propagation of distribution, error-in-variables, Bayesian analysis

## Abstract

Thermal management is a key issue for the downsizing of electronic components in order to optimise their performance. These devices incorporate more and more nanostructured materials, such as thin films or nanowires, requiring measurement techniques suitable to characterise thermal properties at the nanoscale, such as Scanning Thermal Microscopy (SThM). In active mode, a hot thermoresistive probe scans the sample surface, and its electrical resistance *R* changes as a function of heat transfers between the probe and sample. This paper presents the measurement and calibration protocols developed to perform quantitative and traceable measurements of thermal conductivity *k* using the SThM technique, provided that the heat transfer conditions between calibration and measurement are identical, i.e., diffusive thermal regime for this study. Calibration samples with a known *k* measured at the macroscale are used to establish the calibration curve linking the variation of *R* to *k*. A complete assessment of uncertainty (influencing factors and computational techniques) is detailed for both the calibration parameters and the estimated *k* value. Outcome analysis shows that quantitative measurements of thermal conductivity with SThM (with an uncertainty value of 10%) are limited to materials with low thermal conductivity ($k<10\;W\;m^{-1}\;K^{-1}$).

## 1. Introduction

The issues of thermal management in electronic devices (cell phones, laptops, batteries…) became more and more important with the progressive miniaturisation of components observed during the last decades. Understanding heat transfer processes in the corresponding nanostructured materials and devices requires accurate knowledge of the thermal properties of the materials used at the nanoscale. To meet these needs, several optical and near-field techniques have been developed to obtain local thermal information on a small scale [1]. Among these measurement methods, the Scanning Thermal Microscopy (SThM) [2,3] is the most used technique as it enables to reach a lateral resolution less than 100 nm with appropriate probes [4,5], whereas optical approaches such as photothermal radiometry [6], thermo-reflectance [7], and photo-reflectance [8] are limited by light diffraction and have thus higher lateral spatial resolution. Based on conventional atomic force microscopy (AFM) equipped with a miniaturised thermal sensor, SThM devices have been developed actively since the 1990s in order to operate either in passive mode for surface temperature measurements [9] or in active mode for phase transition detection [10], thermal contact resistance [11], and thermal conductivity contrast imaging [12,13,14].

SThM is, in addition, a promising technique for performing quantitative measurements of thermal conductivity at the nanoscale [15]. During the last decade, abundance experimental measurements have been performed with the SThM technique to quantify the thermal properties of nanoscale structures. For example, the thermal conductivity of nanowires embedded in a matrix was estimated [16,17]. SThM has also been used to measure the thermal conductivity of graphene oxide nanosheets [18] or ZnO thin films [14,19]. Unfortunately, most of the results in the literature are given without associated uncertainty or with uncertainties corresponding only to the range or the standard deviation [20] of multiple measurements, and are not traceable to the International System of Units (SI).

The objective of this paper is to provide complete guidance for the calibration of SThM, for traceable estimation of the thermal conductivity *k* at nanoscale and the evaluation of its associated uncertainty using the SThM apparatus, which is a new contribution in this field. This assessment is not trivial because the measurement of the thermal conductivity *k* of a sample by SThM technique is an indirect measurement method [21] which can be represented with the following implicit measurement model [21]

(1) $$h(k,Y,Q_{1},\ldots ,Q_{n})=0,$$

where *h* is a function that relates the measurand *k* (the quantity intended to be measured [22]) to an intermediate measurand *Y* linked to the variation of the thermo-electrical response of the probe (such as the electrical resistance, the temperature, and the electromotive force (EMF), depending on the type of probe used), and to other quantities $Q_{i}$, i=1,…,N involved in the measurement. The elements *h*, *Y* and $Q_{i}$ of (1) are described thereafter.

In SThM active mode, the probe acts both as a heater and as a sensor. In steady state, the hot probe exchanges a constant heat flow corresponding to a $G_{Tot}$ thermal conductance (reciprocal of the thermal resistance) with its surroundings. In air, when the probe is far from the sample, the heat flux dissipates only through the cantilever and the air. When the probe is in contact with a sample, an additional channel is opened for the heat flux to the sample. This thermal flux is characterised by a thermal conductance $G_{sample}$ and is function of the thermal conductivity *k* of the sample among other influencing parameters. The variation *Y* of the thermo-electrical response of the probe between the measurements performed far from the sample and measurements performed in contact with the sample is representative of this thermal conductance $G_{sample}$ and by the way of *k* of the sample. Two steps can then be followed in order to determine the measurement function *h* in (1) linking the intermediate measurand *Y* to the measurand *k* and the $Q_{i}$.

The most challenging way is to build a complete theoretical physical model describing the heat transfer between the probe and the sample that remains complex and involves many influencing quantities [15]. The easiest way that is used in this paper is to build a calibration curve [23,24,25,26,27], based on the measurements of a set of bulk calibration materials with well-known thermal conductivities, and to fit the experimental points with a model deduced from phenomenological studies of the measurements [27,28]. The use of this model and of the calibration curve is based on a strong assumption: the heat transfer conditions are the same during calibration on bulk calibration materials as during measurement on the studied material. The advantage of this method is that it can ensure measurement traceability, provided that the thermal conductivity measurements of the calibration materials are themselves traceable. The International Vocabulary of Metrology (VIM) defines metrological traceability as the “property of a measurement result whereby the result can be related to a reference through a documented, unbroken chain of calibrations, each contributing to the measurement uncertainty” [22].

The evaluation of the uncertainty associated wih the estimated value of *k* requires the evaluation of uncertainties associated with $\left(Y,Q_{1},\ldots ,Q_{n}\right)$ and the inversion procedure. Due to the absence of an explicit relationship between the measurand *k* and the uncertainty sources $\left(Y,Q_{1},\ldots ,Q_{n}\right)$, it is challenging to perform standard uncertainty evaluation using the traditional law of propagation of uncertainties [29]. One solution is to apply Bayesian statistical inversion in order to both predict the value $k^{*}$ of *k* corresponding to an observed value $y^{*}$ of *Y* and estimate the parameters of the SThM calibration curve and their associated uncertainties. The interest of this approach is the joint estimation of the calibration parameters and the thermal conductivities from fresh SThM measurements in addition to the calibration data. The marginal posterior distribution $\pi (k^{*}|y,y^{*})$ can be considered as the measurement result, from which can be extracted point estimates, standard uncertainties, and 95% credibility intervals.

Previous works performed at LNE (Laboratoire National de Métrologie et d’Essais) [30,31] present measurement and calibration procedures with the aim of performing quantitative and traceable measurements of *k* at nanoscale by the SThM technique. A measurement protocol named “dark mode”, where the laser of the optical force detection system of the SThM is switched off, has been proposed to avoid the bias induced by the overheating of the thermoresistive probe due to the laser beam [31]. An analysis of the measurement process and the uncertainty associated with the intermediate measure (corresponding to the electrical resistance variation ΔR of the probe) have been described in detail [30]. Then, a new intermediate measurand Y was defined, and the measurement and calibration protocols were improved in order to build the calibration curve (using a theoretical model and experimental measurements on bulk materials) to ensure the traceability to the International System of Units (SI) of measurements at nanoscale.

**Figure 1:**
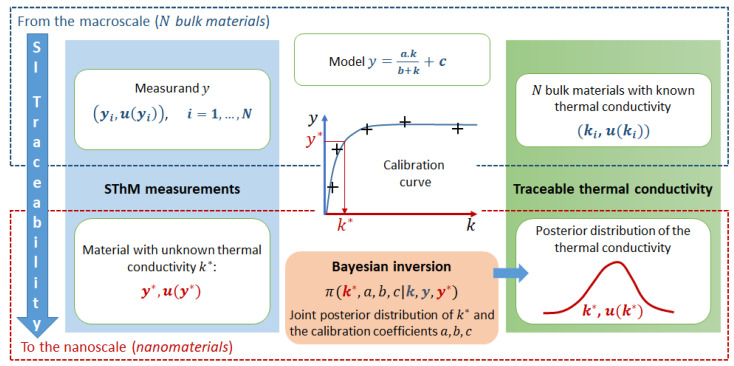
Representation of the workflow for establishing traceability to the SI for thermal conductivity measurements by SThM technique for measurements in diffusive thermal regime.

Based on Bayesian inversion, this paper presents the first complete uncertainty assessment for calibration of SThM apparatus for thermal conductivity *k* measurements. It details the measurement, calibration, and uncertainty assessment processes at the macro-scale and investigates the determination of thermal conductivity at the nano-scale, as well as the assessment of the corresponding measurement uncertainty as illustrated in Figure 1.

## 2. Materials and Methods

SThM is an atomic force microscope (AFM) with an instrumented probe acting as a thermal sensor. Therefore, SThM offers the possibility to characterise surfaces at the nanometric scale in terms of topography, such as standard AFM, as well as in terms of thermal properties. In order to perform thermal conductivity measurements, the probe acts both as a sensor and as a heater. When the probe maps the surface of a sample, its electrical resistance changes as a function of its own temperature, depending on, *inter alia*, heat transfer between the probe and the sample. In this section, the equipment, the used resistive probes, the SThM measurement protocol, the calibration protocol elaborated to perform SI traceable measurement, and the uncertainty assessment method are presented.

### 2.1. Measurement Equipment

#### 2.1.1. SThM System

The LNE’s scanning thermal microscope is a commercial NTEGRA scanning probe microscope from the NT-MDT company. The dedicated Nova-Px software-v2013 enables accurate displacements of sample against the probe using x, y, and z directions of piezoelectric scanners. The contact force between the probe and the sample is controlled using a classical optical feedback system that includes a laser diode beam reflected on the cantilever and a quadrant of photodiodes. In addition to the standard deflection signal of the laser beam, the oscilloscopes included in the software enable the recording of signals from the thermal unit as current intensity or voltages.

#### 2.1.2. Resistance Temperature Probe

**Figure 2:**
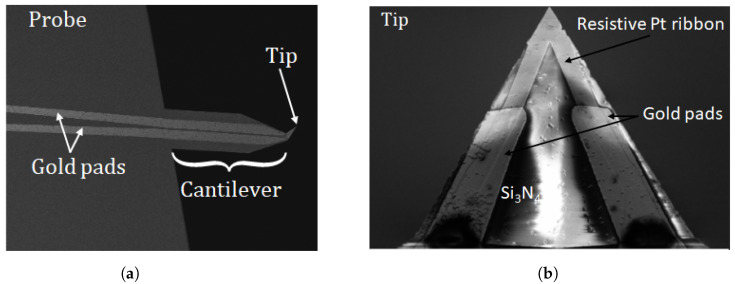
SEM images of a KNT probe (2an type): (**a**) View of the SiN cantilever with the two gold pads (**b**) Zoom on the tip with the resistive Pt ribbon at deposited at the top end of the tip.

The resistance temperature probes used for this study are constituted from a SiN-grooved cantilever with gold pads. A fine resistive ribbon of palladium (about 150 μm length) is deposited on the tip of the cantilever, as illustrated in Figure 2. These probes, called KNT probes, are provided by Kelvin Nanotechnology. The new generation of KNT probes has a nominal electrical resistance $R(T_{ambient})$ in the range of 340 Ω to 450 Ω (measurements performed on a set of 12 probes).

#### 2.1.3. Thermal Unit

**Figure 3:**
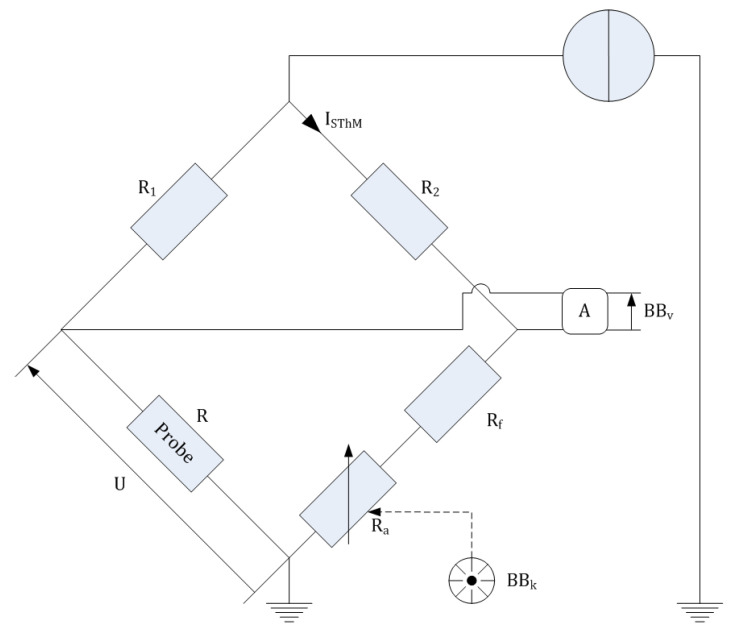
Scheme of the thermal unit encloses an adjustable current generator and a Wheatstone bridge composed of two fixed resistances ($R_{1}$ and $R_{2}$), two adjustable resistances: $R_{f}$ for coarse adjustments and $R_{a}$ for fine adjustments, the resistance temperature probe R and an amplifier setup A to amplify the bridge balance $BB_{v}$ voltage. The probe electrical resistance *R* is included in one leg of the Wheatstone bridge and the adjustable resistance $R_{a}$ in the opposite leg. $R_{a}$ can be set manually by rotary knob arbitrarily scalable in 1000 graduations. Value of the knob adjustment is denoted $BB_{k}$.

The probe is included in a homemade thermal unit that encloses a current generator whose amplitude $I_{SThM}$ extends from 750 µA to 1350 µA and a Wheatstone bridge that is a well-established technique for accurate electrical resistance measurements. Figure 3 describes the homemade thermal unit with a scheme of the Wheatstone bridge adapted to the KNT resistance temperature probes. One leg of the bridge is comprised of the SThM resistance temperature probe in series with a fixed precision resistor $R_{1}$. The other leg is comprised of an adjustable resistance $R_{a}$ in series with two precision resistors $R_{2}$ and $R_{f}$. The precision resistor $R_{2}$ is fixed, and $R_{f}$ is a calibrated decade resistor (DB62-11K from IET Labs) with a Kelvin type 4-terminal configuration. The value of this resistor is adjusted to the lower decade of the nominal electrical resistance $R(T_{ambient})$ for each probe (as an example, for a resistance temperature probe with a nominal electrical resistance of 412 Ω, value of $R_{f}$ is adjusted to 410 Ω). The value of the adjustable resistance $R_{a}$ is manually monitored by a rotary knob arbitrarily scalable in 1000 graduations; the level of graduation for the knob is denoted as $BB_{k}$ in the following. The bridge balance voltage $BB_{v}$ is amplified by a factor A (110). Once $R_{a}$ is adjusted to meet the following equality:

(2) $$R\;\cdot \;R_{2}=R_{1}\;\cdot \;\left[R_{a}+R_{f}\right],$$

the Wheatstone bridge is balanced, and the measured bridge balance value $BB_{v}$, recorded by an external voltmeter, equals zero. An external voltmeter is used rather than the oscilloscope of the Nova-Px software-v2013 because an offset of 58.2 mV (amplified value by a factor 110) on the oscilloscope measurements when the bridge is balanced has been identified. This offset is due to parasitic resistance (the unequal resistance of the wires). In addition to the $BB_{v}$ voltage data recording, an external voltmeter enables the measurement of the probe voltage *U*. A complete description of the SThM setup can be found in paper [30]. Based on the knowledge of the electrical resistances ($R_{a},R_{f},R_{1},R_{2}$) involved in the bridge, the amplificator factor *A*, the measurement of $BB_{v}$ voltage, and the *U* probe voltage, the electrical resistance *R* of the probe can be determined following Equation (3):

(3) $$R=\frac{U}{\frac{R_{a}+R_{f}+R_{2}}{\left(R_{a}+R_{f}\right)\;\cdot \;R_{1}}\left(\frac{R_{2}}{R_{a}+R_{f}+R_{2}}U-\frac{BB_{v}}{A}\right)}.$$

### 2.2. SThM Measurements

#### 2.2.1. Active Mode Configuration

The SThM technique can be used in two different modes: the passive mode, where the probe is cold and comes in contact with the hot sample surface for temperature measurements, and the active mode, dedicated to thermal conductivity measurement, where the probe is hot, self-heated by the Joule effect, and comes in contact with the cold sample surface. In our configuration, we performed measurements in active mode, and our SThM measurement consists of estimating the variation of heat losses from the heated probe to its surrounding environment between the two configurations “out of contact” with the sample and “in contact” with the sample:

- “out of contact” abbreviated in “oc” where the probe is placed far from the thermal influence of the sample. Furthermore, the electrical resistance R of the probe mostly depends on the convective and conductive heat losses between the probe and the ambient air, the current intensity in the probe, the conductive heat losses from the probe to the cantilever, and the heat induced by the laser diode beam illuminating the cantilever. The radiative heat losses can be neglected.
- “in contact” abbreviated in “ic” where the probe is in contact with the sample surface. In this configuration, R depends on the same influencing parameters as in the “out of contact” configuration and on the heat transfers between the probe and the surface sample. These are functions of the thermal properties of the sample and the interface thermal resistance between the probe and the sample.

Bycomparing the probe signals in “oc” and “ic” configurations, a measured quantity value [22] ΔR can be defined associated with the probe used. This measured quantity value ΔR actually corresponds to a temperature drop of the probe between the “oc” and “ic” configurations:

(4) $$\Delta R=R_{oc}-R_{ic}$$

and is, *inter alia* other parameters, function of the thermal conductivity *k* of the sample.

#### 2.2.2. Definition of the Intermediate Measurand

As highlighted in the previous paragraph, the probe electrical resistance R(T) depends on its temperature *T* and follows a quite linear relationship with its temperature for low variation of temperature:

(5) $$R\left(T\right)=R\left(T_{ambient}\right)\;\cdot \;\left[1+\alpha \left(T-T_{ambient}\right)\right],$$

with α the temperature coefficient of the probe material and $R(T_{ambient})$ the electrical resistance of the probe at room temperature. The measured quantity value ΔR is then expressed by:

(6) $$\Delta R=R\left(T_{ambient}\right)\;\cdot \;\alpha \;\cdot \;\left(T_{oc}-T_{ic}\right).$$

Unfortunately, experiments highlight the long-term drift of the value of $R(T_{ambient})$ depending on the ageing of the probe. Indeed, wear of the tip affects the $R(T_{ambient})$ value on many levels. Many landings cause wear of the apex that changes both the size of the solid-solid contact between the tip and the surface of the sample and the length of the resistive Pd ribbon. In addition, a high value of the current intensity can induce matter transport resulting from electron displacement. This can cause the Pd ribbon to be damaged. In metallic thin layers, the electro-migration is prevalent. One way to correct the influence of this drift is to work with a reference sample. By performing measurements on the reference sample before each measurement on a studied sample, we define an intermediate measurand $Y_{m}$ equal to the ratio between $\Delta R_{sample}$ obtained on the studied sample and $\Delta R_{ref}$ obtained on the reference sample:

(7) $$Y_{m}=\frac{\Delta R_{sample}}{\Delta R_{ref}}=\frac{\Delta R_{sample}}{\Delta R_{SiO_{2}f}}=\frac{R_{oc}^{sample}-R_{ic}^{sample}}{R_{oc}^{SiO_{2}f}-R_{ic}^{SiO_{2}f}}.$$

The reference sample must be chemically inert and must remain stable over time. Furthermore, SThM measurements require perfect surface conditions with the sample [14,27]. Finally, the material must have a thermal conductivity within the sensitivity range of the SThM technique, i.e., lower than 10 $W\;m^{-1}\;K^{-1}$ as we will discuss in Section 4.1. The reference sample selected by LNE is a piece of fused amorphous silica $SiO_{2}f$ with a roughness value Ra of 0.56 nm.

In order to evaluate the effect of repeatability and reproducibility conditions with regard to some uncertainty sources, our measurement process is repeated several times in different conditions described in Section 2.2.3. Each condition i=1,…,10 provides a corresponding measurement result $Y_{m,i}$ composed of a measured quantity value $y_{m,i}$ and its associated uncertainty $u(y_{m,i})$. Finally, the intermediate measurand *Y* is computed as the mean ${\bar{Y}}_{m}$ of this set of ten individual measurement results $Y_{m,i}$:

(8) $$Y={\bar{Y}}_{m}=\frac{\overset{10}{\sum_{i=1}}Y_{m,i}}{10}.$$

#### 2.2.3. SThM Measurement Protocol

##### Sample Requirements

The sample dimensions must allow the landing of the SThM probe but be small enough to allow two samples to be inserted at the same time on the SThM sample holder. For this study, typical dimensions (diameter) were 0.5 and 1 cm. The sample must be protected from any effect likely to alter its properties (contamination, dust, oxidation, water contact, temperature changes). The contact between the tip and the sample is assumed to be identical from one sample to another, provided that the scanning surfaces are locally flat [27,32]. The sample surface should be mirror polished to avoid poor mechanical contacts between the tip and the surface and to assume that the contact resistance is the same for all samples.

##### Measuring Conditions

**Figure 4:**
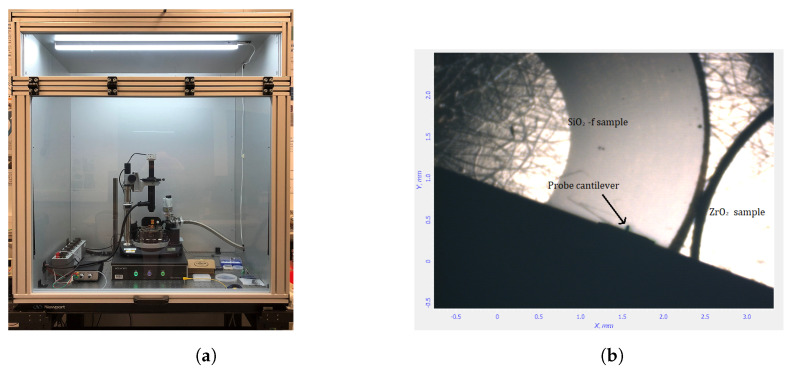
Views of apparatus: (**a**) SThM and thermal unit inside the dedicated enclosure to decrease influence of thermal drifts and (**b**) view of the location of the probe above the $SiO_{2}f$ reference sample. Range of displacement of x, y direction piezoelectric scanners enables to switch from $SiO_{2}f$ reference sample to the studied sample ($ZrO_{2}$ sample for the picture).

Temperature and relative humidity of the environment must be controlled as they influence directly the heat transfer between the tip and the environment. Humidity enhances the water meniscus formed at the tip/sample contact and, consequently, the thermal conductance of the contact [33].The SThM is located in an air-conditioned room where the temperature is set to 21 °C and the relative humidity is 50%. A station sensor located close to the scanning probe microscope monitors the environment. The variations are very slow over a day or a week, less than 0.5 °C and 3%, respectively. These conditions limit the influence of the hydrophilic nature of the different samples. The measurements were performed in air with thermal steady-state conditions. Some thermal drifts have been measured when performing measurements with the NTEGRA protection cover, which is quite airtight [30]. These thermal drifts are due to air conditioning and SThM heat sources such as step motors and piezo motors. A specific homemade enclosure viewed in Figure 4a has been developed to protect both the SThM and the thermal unit from air conditioning disruptions and to limit thermal drifts from internal heat sources.

Two samples are placed at the same time on the SThM sample holder, as illustrated in Figure 4b: the reference sample (fused silicon dioxide ($SiO_{2}f$) and the studied sample. The two samples are close enough to assume that they are at the same temperature. The NTEGRA x, y directions of piezoelectric scanners provide displacement of the sample holder to place either of the samples under the probe.

Measurements require thermal steady-state conditions. Samples stay in the SThM for a stabilisation time of at least 2 h before starting measurements. To avoid any laser effects on bridge balance voltage $BB_{v}$ (probe overheating, thermal drift), measurements are performed in “dark mode” [31].

In dark mode, the laser of the probe guiding system is turned off to avoid continuous heating of the probe. The probe landing on the sample surface was performed step-by-step by the user. The contact of the probe with the sample surface is indicated by a strong discontinuity in the $BB_{v}$ voltage signal. The “oc” distance between probe and sample was 1000 steps (approximately 1.5 mm) during these experiments. This chosen distance of 1.5 mm is large enough to avoid the thermal influence of the sample in the dark mode.

##### Measurement Process

The measurements were performed with direct current (DC) heating of the resistive probe in a “constant current” configuration. At the beginning of the experiments, the value of the electrical current $I_{SThM}$ is fixed arbitrarily to 900 µA. The value of the electrical current is maintained below the critical value specified by the supplier ($I_{SThM}<2.5\;\mathrm{mA}$) in order to avoid damage or premature wear of the probe. In addition, a study of the influence of the value of the electrical current showed that working with an electrical current of 900mA provided the best measurement repeatability (<$2.10^{-4}\;a.u.$) for the intermediate measurand *Y* [34]. The bridge balance is balanced by adjusting the value of $R_{a}$ with the rotary knob $BB_{k}$, this adjustment of the $BB_{k}$ is strictly kept constant during all of the calibration experiments. For the preliminary adjustments of $I_{SThM}$ and $BB_{v}$, the probe is in an “out of contact” configuration.

After these preliminary settings, the experiment sequence for each measurement is:

- after stabilisation (criteria of standard deviation $<10^{-4}\;V$ for $BB_{v,oc}^{ref}$ mean value calculated with measurements performed during a 100 s period), start recording of $BB_{v,oc}^{ref}$ and $U_{oc}^{ref}$ signals during a 100 s period with the probe in an “out of contact” configuration above the reference $SiO_{2}f$ sample;
- land with “dark mode” on one position of the $SiO_{2}f$ reference sample, wait for stabilisation (with the same criteria as for the first step), record $BB_{v,ic}^{ref}$ and $U_{ic}^{ref}$ signals during a 100 s period with the probe in an “in contact” configuration;
- remove the probe from contact and wait for stabilisation (with the same criteria as for the first step), record $BB_{v,ic}^{ref}$ and $U_{ic}^{ref}$ signals during a 100 s period with the probe in “out of contact” configuration; repeat these three operations for two other landings at the same position above and on the $SiO_{2}f$ reference sample (repeatability of measurements).
- After 3 measurements at the same position on the $SiO_{2}f$ reference sample, from “out of contact” configuration, move to another position above the sample (reproducibility measurements). After stabilisation (criteria of standard deviation $<10^{-4}\;V$ for $BB_{v,oc}^{ref}$ mean value calculated with measurements performed during a 100 s period), start recording of $BB_{v,oc}^{ref}$ and $U_{oc}^{ref}$ signals during a 100 s period with the probe in “out of contact” configuration for the new position above the reference $SiO_{2}f$ sample;
- land with “dark mode” on the new position of the $SiO_{2}f$ reference sample, wait for stabilisation (with the same criteria as for the first step), record $BB_{v,ic}^{ref}$ and $U_{ic}^{ref}$ signals during a 100 s period with the probe in “in contact” configuration;
- remove the probe out of contact, waiting for stabilisation (with the same criteria as for the first step), record $BB_{v,ic}^{ref}$ and $U_{ic}^{ref}$ signals during a 100 s period with the probe in “out of contact” configuration;
- from “out of contact” configuration, move to another position above the sample, and repeat the two steps described in the two last bullets for this third location on the sample.
- After measurements on the $SiO_{2}f$ reference sample, perform measurements on the studied sample following the same protocol as for the $SiO_{2}f$ reference sample.

**Figure 5:**
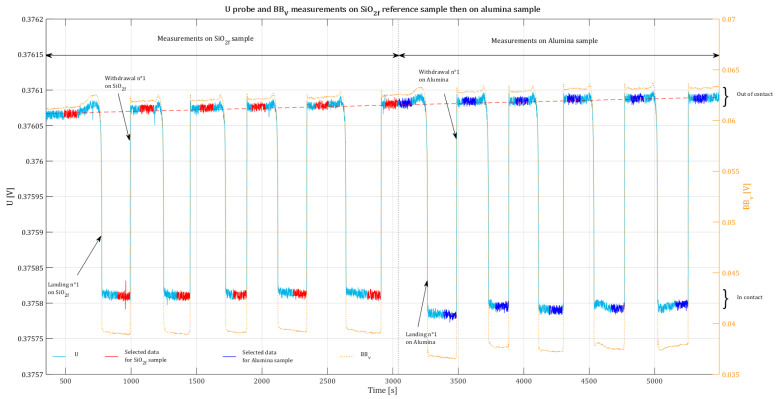
Work flow of measurement sequence of *U* probe (cyan solid line) and $BB_{v}$ (orange dotted line) signals: five successive landings on the $SiO_{2}f$ reference sample following by five successive landings on the studied sample (alumina sample in this example). For each sample (reference and studied) the first three landings are performed on the same location and the last two on different locations. Measurement values used for *R* computation are identified in red for the $SiO_{2}f$ reference sample and in blue for the studied sample. The residual drift is highlighted with the red dashed line for *U* “out of contact” measurements.

An example of the experimental *U* and $BB_{v}$ signal recording is presented in Figure 5. The five steps on the curve correspond to the measurements on the $SiO_{2}f$ reference sample, and the last five steps correspond to the measurements on the studied alumina calibration sample.

We obtain eleven $BB_{v}^{ref}$ and eleven $U^{ref}$ measurement data (five in “in contact” configuration and six in “out of contact” configuration) on a $SiO_{2}f$ reference sample associated with, respectively, eleven $BB_{v}^{sample}$ and eleven $U^{sample}$ measurement data on the studied sample. Using this measurement data, for each sample (reference and studied samples), we calculate eleven electrical resistance values for the probe following Equation (3) (five in “in contact” configuration and six in “out of contact” configuration). Then, we determine ten resistance differences per sample as defined in Equation (4). A regular residual drift is observed both on *U* and $BB_{v}$ measurements, mainly visible for “out of contact” data (represented by the red dashed line in Figure 5). This residual drift is induced by the electronics of the SThM (i.e., the motors of the piezoelectric stage). In order to reduce the influence of this drift of signals, we compute the $y_{m,i}$ (*i* from 1 to 10) measured quantity values from each individual data in a chronological way ($U_{ref}$ and $BB_{v,ref}$ data from the first landing on the reference sample associated to $U_{sample}$ and $BB_{v,sample}$ data from the first landing on the studied sample and so one for next data. By this way, we obtain ten measurement results $Y_{m,i}$, which are used to calculate the resulting *Y* intermediate measurand as the mean value following Equation (8).

To check the potential influence of landing or withdrawal conditions, we identified two types of resistance differences. The first one, $\Delta R_{landing}$, identified as “landing”, corresponds to the resistance difference between measurements performed in the “out of contact” condition before the contact and the measurements performed during the “in contact” configuration. The second one, $\Delta R_{withdrawal}$, identified as “withdrawal”, corresponds to the resistance difference between measurements performed in the “out of contact” condition after the contact and the measurements performed during the “in contact” configuration.

(9) $$\Delta R_{landing}=R_{oc,Before\;Contact}-R_{ic}$$

(10) $$\Delta R_{withdrawal}=R_{oc,After\;Contact}-R_{ic}$$

In conclusion, we compute ten measured quantity values $y_{m,i}$ for the intermediate measurand *Y* with repeatability and reproducibility conditions. The first six successive measurements. $y_{m,i}$ with *i* from one to six, are performed on the same location; the last four measurements, $y_{m,i}$ with *i* from seven to ten, are performed on two other locations in order to evaluate the potential influence of heterogeneity of the sample (reproducibility conditions). In addition, we also studied the potential influence of landing measurement conditions associated with odd *i* indexes and withdrawal measurement conditions associated with even *i* indexes. Measurement conditions and identification are summarized in Table 1.

### 2.3. SThM Calibration Protocol

Two ways are available in order to link the intermediate measurand *Y* to the thermal conductivity of the studied sample. The first solution is to establish a physical model describing heat transfers between the probe and the surface sample. Unfortunately, it is challenging to develop rigorous physical models at the nanoscale because the values of the influencing parameters are difficult to evaluate at these scales and not always reliable. Another solution is to establish a calibration curve based on materials with thermal conductivities measured at the macroscopic scale [23,24,27,28,35,36,37]. However, this method is based on a strong assumption, as highlighted in Section 1, that the heat transfer conditions are the same during both the calibration and sample measurement steps. Since calibration is performed at the macroscopic scale, heat transfers are diffusive. Particular care must be taken when measuring nanomaterials with a mean free path higher than the size of the studied structure (involving ballistic heat transfers).

The calibration function is mainly based on phenomenological study, and the calibration parameters are identified with Bayesian inversion from the experimental values and the mathematical model.

#### 2.3.1. Definition of the Calibration Model

Based on phenomenological studies [24,28,38], a mathematical link has been established between the variation of the probe electrical resistance when it is put in contact with the material and the thermal conductivity of the material. These studies are based on the heat transfer from the hot probe (heated by the Joule effect) to the “cold” sample.

The thermal conductances involved in the probe-sample system during measurements are the thermal conductance relating to the probe heat losses through convection and radiation $G_{env}$, the thermal conductance relating to the probe heat transfer through the cantilever $G_{cant}$, the effective thermal conductance of the thermal contact between the probe and the sample (solid-solid thermal conduction, air and water meniscus thermal conduction $G_{contact}$ and the thermal conductance of the sample $G_{sample}$.

During “out of contact” measurement, the total thermal conductance $G_{tot,oc}$ of the probe-sample system is:

(11) $$G_{tot,oc}=G_{env,oc}+G_{cant}.$$

During “in contact” measurement, the total thermal conductance $G_{tot,ic}$ of the probe-sample system is:

(12) $$G_{tot,ic}=G_{env,ic}+G_{cant}+\frac{G_{contact}.G_{sample}}{G_{contact}+G_{sample}}.$$

The variation of the thermal conductance of the probe-sample system between “oc” and “ic” configurations is consequently:

(13) $$G_{tot,ic}-G_{tot,oc}=\frac{G_{contact}.G_{sample}}{G_{contact}+G_{sample}}+\left(G_{env,ic}-G_{env,oc}\right).$$

Assuming that heat flow in the sample is diffusive, that means that the mean-free path Λ of the energy carriers, defined as the average distance between two successive inelastic shocks of energy carriers, is lower than the radius *b* of the thermal contact between the probe and the sample; the thermal conductance $G_{sample}$ is given by:

(14) $$G_{sample}=4\beta kb$$

with β a specific coefficient corresponding to the influence of the shape of the thermal contact. As a result, the variation of thermal conductance $\Delta G_{tot}$ can be written as:

(15) $$\Delta G_{tot}=\frac{G_{contact}\;\cdot \;k}{\frac{G_{contact}}{4\beta kb}+k}+\Delta G_{env}$$

Therefore, the variation of the electrical resistance of the probe between “oc” measurement and “ic” measurement can be described as:

(16) $$R_{oc}-R_{ic}=\frac{a_{R}k}{b_{R}+k}+c_{R}$$

where $a_{R}$, $b_{R}$ and $c_{R}$ are parameters relative to the different thermal conductances involved during measurements. As $R_{oc}^{SiO_{2}f}-R_{ic}^{SiO_{2}f}$ does not depend on sample thermophysical properties, the *k* dependence of $\frac{\Delta R_{Sample}}{\Delta R_{SiO_{2}f}}$ remained unchanged when divided by a constant value. The calibration law of the intermediate measurand *Y* obeys thus the following law:

(17) $$Y=\frac{\Delta R_{Sample}}{\Delta R_{SiO_{2}f}}=\frac{ak}{b+k}+c$$

Parameters *a*, *b* and *c* are estimated using Bayesian identification in order to estimate the uncertainty associated with these coefficients, which take into account the uncertainty associated with both the thermal conductivity of known materials and the intermediate measurand considered in this paper.

A representation of the calibration curve is provided in Figure 1, and the corresponding calibration model is identified as Equation (17).

#### 2.3.2. Calibration Materials

Drawing on the results from the Quantiheat project [32], the choice of the calibration samples was based on their thermophysical properties, assuming that the thermal conductivity measured at the macroscale and at the nanoscale are comparable in terms of their mechanical stability and low roughness. To ensure the traceability of measurements, the materials have been chosen so that their thermal conductivity can be measured using traceable techniques. This requires them to be homogeneous and isotropic. Twelve samples of twelve different bulk materials (polymers, ceramics, and pure metals) with thermal conductivity k ranging from 0.1 $W\;m^{-1}\;K^{-1}$ to 100 $W\;m^{-1}\;K^{-1}$ have been selected for this study: poly(methyl methacrylate) (PMMA), poly-oxymethylene in copolymer (POM-C), borosilicate glass, two different grades of fused silicon dioxide ($SiO_{2}f$) and ($SiO_{2}-NEGS1$), zircon oxide ($ZrO_{2}$), titanium oxide ($TiO_{2}$), alumina (poly crystal aluminium oxide $Al_{2}O_{3}p$), sapphire ($\alpha -Al_{2}O_{3}$), germanium (Ge), p doped silicon ($Sip^{\;++}$), and zinc (Zn). All samples were mirror-polished to minimise roughness. The polymers were prepared by ultramicrotomy (cryogenic cutting). Table 2 presents materials, dimensions, structures, providers, measured thermal conductivity, and measured roughness of selected materials. Each sample is in the shape of a disc with a 10mm diameter, except for the two polymer samples, which have a flat surface prepared by ultramicrotomy of around $1\;{\mathrm{mm}}^{2}$.

The thermal conductivity of each material was determined at 23 °C on bulk specimens (a few mm thick) using an indirect and traceable method. This method is based on the measurements of the thermal diffusivity by the laser flash method [39,40], the specific heat by differential scanning calorimetry, and the density by the Archimedean method. The expanded uncertainty associated with the measurement of thermal conductivity by this indirect method has been estimated at 5% [41].

### 2.4. Method for the Evaluation of the Uncertainty Associated with the Estimation of the Intermediate Measurand

The intermediate measurand *Y* considered in this paper is the ratio of the resistance difference for the tested sample between the “ic” and “oc” configurations and the same difference obtained for the reference sample $SiO_{2}f$ (see Equation (8)). In order to evaluate dedicated uncertainty from repeatability and reproducibility conditions, *Y* is obtained as the combination of individual quantity measured values $y_{m}$ and their associated uncertainties. Based on the protocol described in Section 2.2.3, we obtained ten $y_{m,i}$ values for various conditions as described in Table 1. Some of these values are obtained from repeatability conditions (measurements at the same location), and others from reproducibility conditions (measurements at different locations or with two different configurations: landing or withdrawal conditions). In this way, we study the individual contribution of each uncertainty source (instrumental, repeatability, and reproducibility) in Section 3.2.

**Figure 6:**
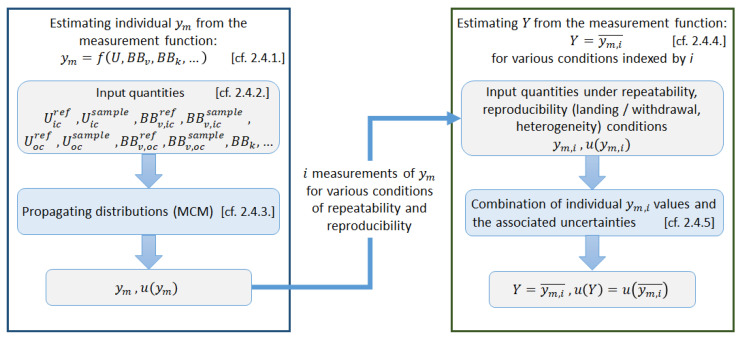
Main steps for the evaluation of measurement uncertainty: In a first step: Modelling the measurement process for $Y_{m}$ from Section 2.4.1, Evaluating input quantities with associated Probability Distribution Functions for $Y_{m}$ in Section 2.4.2, Propagating with Monte Carlo method (MCM) in Section 2.4.3 to report single quantity measured values $y_{m}$ with its associated uncertainty $u(y_{m})$. In a second step: Use $y_{m,i}$ input quantities measured under various conditions indexed by *i* (*i* from 1 to 10) with measurement model described in Section 2.2.2 then combine the ten $y_{m,i}$ measurements and the associated uncertainties in Section 2.4.4 to report the $\bar{y}$ mean value with standard uncertainty $u(\bar{y})$.

The estimated value of the intermediate measurand *Y* and its associated uncertainty include repeatability and reproducibility conditions influences. The different steps in the evaluation of the measurement uncertainty associated with *Y* are presented in Figure 6.

The following sections (Section 2.4.1, Section 2.4.2 and Section 2.4.3) describe the evaluation of measurement uncertainty on each individual measurand $Y_{m}$, based on works (calculation, procedures, and conclusions) performed previously at LNE by Ramiandrisoa et al. [30], in which the evaluation of the measurement uncertainty associated with a single ΔR was considered. Then the next section (Section 2.4.4) describes the combination of $y_{m,i}$ measurements and uncertainty to report the intermediate measurand *Y* value for various conditions with associated standard uncertainty.

#### 2.4.1. Modelling the Measurement Process for Individual Measurand

Four resistance measurements are involved in the mathematical model of the individual measurand $Y_{m}$ value as described in (7), each being determined thanks to the Wheatstone bridge in Figure 3. As a result, the expression for one of these four resistances (denoted as *j* from 1 to 4) is:

(18) $$R_{j}=\frac{U_{j}}{\frac{R_{v}+R_{2}}{R_{v}R_{1}}\left(\frac{R_{2}}{R_{v}+R_{2}}U_{j}-\frac{BB_{j}}{A}\right)}$$

$U_{j}$ is the voltage (in V), $BB_{j}$ is the bridge balance (in V), $R_{1}$ is a fixed resistance of the bridge, given by the technical specifications (in Ω), $R_{2}$ is a fixed resistance of the bridge, given by the technical specifications (in Ω),

(19) $$A=\frac{R_{10k}}{R_{1k}}\frac{R_{1k}^{\prime }+R_{10k}^{\prime }}{R_{1k}^{\prime \prime }}$$

is the amplification factor of the electrical chain (in Ω) and

(20) $$R_{v}=R_{f}+R_{v,min}+\frac{BBk-BBk_{min}}{BBk_{max}-BBk_{min}}\left(R_{v,max}-R_{v,min}\right)$$

is the variable resistance (in Ω). BBk=125 denotes the graduation of the know, with minimum and maximum graduations respectively denoted as $BBk_{min}=0.5$ and $BBk_{max}=1003$. All three quantities have a negligible contribution to the uncertainty and are consequently considered as fixed.

#### 2.4.2. Evaluating Input Quantities for Individual Measurand

In order to perform the Monte Carlo simulation, it is required to assign a Probability Density Function (PDF) to each input quantity in the mathematical model. The following subsections describe the choice made for every single input quantity.

**Voltages:**

The voltage measurements are denoted as *U* and $BB_{v}$ in Figure 3. Two 34401A multimeters measured one *U* voltage and the other one $BB_{v}$ voltage. Three errors are considered to be the most influential ones: the trueness, the quantification, and the repeatability.

- Trueness of the multimeters: This error is the same for each measurement of a voltage, whether the sample is in or out of contact, and whether the unknown sample or the reference sample is measured, but is specific for each multimeter. Available information about the trueness error comes from the calibration certificate of each multimeter. These calibration certificates provide trueness corrections $U_{true}$ and $BB_{v,true}$ with an associated expanded uncertainty $U\left(U_{true}\right)=U\left(BB_{v,true}\right)=2.5$ μV, using a coverage factor k=2. This correction is applied to the measurements, and a Gaussian probability distribution is assigned with a zero mean and
  (21) $$u\left(U_{true}\right)=\frac{U\left(U_{true}\right)}{k}=1.25\times 10^{-6}\;V$$
  and
  (22) $$u\left(BB_{v,true}\right)=\frac{U\left(BB_{v,true}\right)}{k}=1.25\times 10^{-6}\;V$$
  as standard deviations.
- Quantification of the multimeters: The multimeters have the same quantification step q=1 μV in the studied range. As a consequence, the quantification error lies in the interval $\left[-5\times 10^{-7};5\times 10^{-7}\right]$. A rectangular probability distribution is assigned. However, this (unknown) error may be different for each voltage measurement. As a result, we define a different input quantity for each different voltage measurement.
- Repeatability: In order to evaluate the repeatability of the voltage measurement, our measurement corresponds to the mean values $\bar{U}$ and $\bar{BB_{v}}$ of the respective *U* voltage and $BB_{v}$ voltage for 100 measuring points (corresponding to a period of 100 s) associated with their respective standard deviations.
- Measurement model for voltages: As a result, the measurement model used for each voltage measurement (in contact/out of contact) is:
  (23) $${\bar{U}}_{i}=U_{true}+U_{iq}+U_{iR}$$
  and
  (24) $${\bar{BB}}_{v,i}=BB_{v,true}+BB_{v,iq}+BB_{v,iR}$$
  where *i* is either equal to ic for the “in contact” voltage or to oc for the “out of contact” voltage, $U_{true}$ and $BB_{v,true}$ are the trueness corrections, $U_{iq}$ and $BB_{v,iq}$ are the quantification errors and, $U_{iR}$ and $BB_{v,iR}$ are the repeatability errors. An example of corresponding parameters are summarised in Table 3 for the PMMA sample.

**Resistances involved in the Wheatstone bridge**

In the mathematical model of the measurement process, 9 resistances are involved ($R_{1}$, $R_{2}$, $R_{f}$, $R_{v,max}$, $R_{10k}$, $R_{1k}$, $R_{1k^{\prime }}$, $R_{1k}^{\prime \prime }$ and $R_{10k}^{\prime }$). In this section, we present the general mathematical model used to evaluate the uncertainty associated with a resistance, and we provide the summary for each considered resistance.

A resistance *R* is obtained as a reading with an associated trueness error, with a dilatation correction factor taking into account the difference between the temperature $T_{L}$ in the laboratory and the reference temperature $T_{ref}=25$ °C:

(25) $$R_{i}=R_{0}+TCR\;\cdot \;\left(T_{L}-T_{ref}\right)\;\cdot \;R_{0}=R_{0}+TCR\;\cdot \;\Delta T\;\cdot \;R_{0}$$

The trueness of the resistance is evaluated thanks to the technical specifications: $R_{0}=R_{nom}\;\pm a\;\%$, where $R_{nom}$ is the nominal value of the resistance. As a consequence, $R_{0}$ is assigned a rectangular probability distribution over the interval $\left[R_{nom}-a/100\;\cdot \;R_{nom};R_{nom}-a/100\;\cdot \;R_{nom}\right]$. The Temperature Coefficient Ratio (TCR) is considered a fixed value. Regular measurements of the temperature in the laboratory show that it lies between 20.3 °C and 20.5 °C. As a consequence, ΔT is assigned a rectangular probability distribution over the interval [−4.5;−4.7].

In addition to the known resistances involved in the mathematical process, there is a parasitic resistance $R_{parasitic}$ that is the sum of the parasitic resistances of the probe $R_{pprobe}$ and that of the bridge $R_{pbridge}$. This latter can be decomposed as the sum uncertainty sources: the reproducibility $R_{pbR}$ and the quantification error $R_{pbq}$:

(26) $$R_{parasitic}=R_{pprobe}+R_{pbridge}=R_{pprobe}+R_{pbR}+R_{pbq}$$

However, they are not considered in our mathematical model as they do not have any significant contribution to the uncertainty of the intermediate measurand when we consider the SThM indication to be $\frac{\Delta R_{Sample}}{\Delta R_{SiO_{2}f}}$.

An example of assigning probability distributions to all input quantities involved in $y_{m}$ measurement process is given in Table 3 in Section 3.1.

#### 2.4.3. Propagating Distributions for Individual Measurand

The evaluation of associated uncertainty $y_{m}$ measurement is performed using the Monte Carlo method (MCM), according to the principles of Supplement 1 to the GUM (Guide to the Expression of Uncertainty in Measurement) [22,42]. The previous quantification of the input quantities, described in Section 2.4.2, consisted in the choice of suitable Probability Distribution Functions (PDFs) based on the work of Ramiandrisoa [30]. Implementation of the propagation of these distributions is performed using LNE-MCM software-v2017 [43], with $M=10^{6}$ simulations in order to get an overview of the PDFs for $y_{m}$ measured quantity value (with a best estimate, a standard uncertainty, and a coverage interval).

As a result, each individual measured quantity value $y_{m}$ is characterised, and the associated uncertainty of individual $y_{m}$ is evaluated.

#### 2.4.4. Combining Reproducibility Measurements

In this study, the reproducibility measurements ${\left(y_{m,i},u\left(y_{m,i}\right)\right)}_{i=1,\ldots ,10}$ are combined using the random effects model defined as

(27) $$y_{m,i}=\mu +\lambda_{i}+\varepsilon_{i}$$

where μ is the overall arithmetic mean response, the ${\left\{\lambda_{i}\right\}}_{i=1,\ldots ,10}$ are the effects of the reproducibility conditions (which are assumed to have a Gaussian distribution with mean 0 and standard deviation τ), the ${\left\{\varepsilon_{i}\right\}}_{i=1,\ldots ,10}$ are random effects assumed Gaussian with mean 0 and standard deviation the reported standard uncertainties ${\left\{u\left(y_{m,i}\right)\right\}}_{i=1,\ldots ,10}$, the $\lambda_{i}$ and the $\varepsilon_{i}$ are assumed to be independent.

Such a model is generally used to combine heterogeneous data, e.g., from interlaboratory studies or meta-analyses, where the $\lambda_{i}$ are referred to as “laboratory effects”. The parameter τ is often called “dark uncertainty” and is used to capture excess variability in the measurements with respect to the reported uncertainties.

In this application, we aim to identify all sources of uncertainty during measurements under both repeatability and reproducibility conditions. We suspect other influencing factors than material heterogeneity or landing/withdrawal conditions. The main suspected factor is the variation in force applied from one measurement to another, which could not be quantified within the scope of this study. In our case, “dark uncertainty” has a physical meaning related to underestimated $u(y_{m,i})$ uncertainties.

In this study, the parameters μ are τ are estimated using the Bayesian approach of [44] with a non informative prior on μ, π(μ)∝1 and the following prior for τ as recommended in the NIST Consensus Builder [44]: $\tau ∼\mathrm{HalfCauchy}(\mathrm{mad}\left({\left\{y_{m,i}\right\}}_{i}\right))$, where mad is the median absolute deviation.

### 2.5. Bayesian Approach to Estimate the Thermal Conductivity from SThM Measurements

#### 2.5.1. Error-in-Variables Representation

In this section, we denote $X=(X_{1},\ldots ,X_{N})$ the vector of random variables modelling the thermal conductivities measured for the bulk materials and $Y=(Y_{1},\ldots ,Y_{N})$ the vector of random variables modelling the corresponding SThM measurements. Due to the normality arising from the GUM uncertainty propagation for both SThM and thermal conductivity measurements, the following error-in-variables representation (a regression model that accounts for measurement errors in the independent variables) is used:

(28) $$\begin{array}{cc} x_{i} & ∼N(X_{i},u\left(x_{i}\right)), \end{array}$$

(29) $$\begin{array}{cc} y_{i} & ∼N(Y_{i},u\left(y_{i}\right)), \end{array}$$

where $x_{i}$ and $y_{i}$ are respectively the reported estimates of the thermal conductivity and the SThM measurement for material *i*, $u(x_{i})$ and $u(y_{i})$ are their associated uncertainties respectively.

The relationship (17) between *Y* and *X* can be expressed as

(30) $$Y_{i}=h_{\theta }\left(X_{i}\right),i=1,\ldots ,N,$$

where $h_{\theta }$ is the calibration curve and θ=(a,b,c).

Denote $X^{*}$ the random variable representing the unknown thermal conductivity of a material under test and $Y^{*}$ the random variable modelling the reported SThM measurement result $y^{*}$ and its associated uncertainty $u(y^{*})$. Similarly to (29) and (30), we have

(31) $$y^{*}∼N(Y^{*},u\left(y^{*}\right)),$$

(32) $$Y^{*}=h_{\theta }\left(X^{*}\right).$$

In the following, we denote μ, the vector containing all the reported SThM measurements

(33) $$\mu =\left(Y^{*},Y_{1},\ldots ,Y_{N}\right)=\left(h_{\theta }\left(X^{*}\right),h_{\theta }\left(X_{1}\right),\ldots ,h_{\theta }\left(X_{N}\right)\right).$$

It is important to note that this approach can be readily extended to multiple (say *M*) new SThM measurements $y^{*}=\left(y_{1}^{*},\ldots ,y_{M}^{*}\right)$ in which case $Y^{*}$ and $X^{*}$ read as the vectors $Y^{*}=\left(Y_{1}^{*},\ldots ,Y_{M}^{*}\right)$ and $X^{*}=\left(X_{1}^{*},\ldots ,X_{M}^{*}\right)$ respectively.

#### 2.5.2. Bayesian Paradigm

In the Bayesian paradigm [45], all quantities involved, namely $X^{*},\theta ,X,Y,Y^{*}$, are viewed as parameters to be jointly updated in the inference process by the information contained in the measurements. It is important to note that, contrary to classical approaches to inversion, the SThM measurements performed on unknown materials $y^{*}$ are used to update knowledge on all parameters and are not only used for the prediction of $X^{*}$. In other words, using a Bayesian approach allows us to simultaneously estimate the parameters θ of the calibration curve and make predictions from the curve.

According to (30), $Y_{i}$ explicitly depends on θ and $X_{i}$, so that its posterior distribution can be obtained as a by-product of the Bayesian analysis from the samples of the joint posterior distribution of θ and $X_{i}$. For this reason, we chose to remove *Y* and $Y^{*}$ from the Bayes formula.

Denoting $x=(x_{1},\ldots ,x_{N})$ and $y=(y^{*},y_{1},\ldots ,y_{N})$, the Bayes formula gives the joint posterior distribution $\pi \left(X^{*},\theta ,X|x,y\right)$ of all the quantities involved as

(34) $$\pi \left(X^{*},\theta ,X|x,y\right)\propto l\left(x,y|X^{*},\theta ,X\right)\pi \left(X^{*},\theta ,X\right),$$

where $l\left(x,y|X^{*},\theta ,X\right)$ is the likelihood of the data and $\pi \left(X^{*},\theta ,X\right)$ is the prior distribution of the parameters.

The overall objective of estimating the thermal conductivity from SThM measurements consists in estimating the so-called marginal posterior distribution of $X^{*}$ given all the measurements which reads $\pi (X^{*}|x,y)$ and is mathematically obtained from (34) as the integral

(35) $$\pi \left(X^{*}|x,y\right)=\int_{\theta }\int_{X}\pi \left(X^{*},\theta ,X|x,y\right)d\theta dX.$$

#### 2.5.3. Likelihood

The likelihood can be factorized as

(36) $$l\left(x,y|X^{*},\theta ,X\right)=l\left(x|X\right)\times l\left(y|X^{*},\theta ,X\right),$$

where $l\left(x|X\right)$ and $l\left(y|X^{*},\theta ,X\right)$ are defined thereafter.

Denoting $\Sigma_{x}$ the covariance matrix of the Gaussian vector *x*, we have $x∼N_{N}\left(X,\Sigma_{x}\right)$ and the associated part of the likelihood writes

(37) $$l\left(x|X\right)\propto \frac{1}{\sqrt{\det\Sigma_{x}}}\exp\left(-\frac{1}{2}{\left(x-X\right)}^{T}\Sigma_{x}^{-1}\left(x-X\right)\right).$$

Denoting $\Sigma_{y}$ the covariance matrix of the Gaussian vector *y*, we have $y∼N_{N+1}\left(Y,\Sigma_{y}\right)$

(38) $$\left(\begin{array}{c} y^{*}\\ y_{1}\\ \vdots \\ y_{N} \end{array}\right)∼N_{N+1}\left(\left(\begin{array}{c} h_{\theta }\left(X^{*}\right)\\ h_{\theta }\left(X_{1}\right)\\ \vdots \\ h_{\theta }\left(X_{N}\right) \end{array}\right),\left(\begin{array}{ccccc} u^{2}\left(y^{*}\right) & 0 & 0 & \cdots & 0\\ 0 & u^{2}\left(y_{1}\right) & 0 & \cdots & 0\\ \vdots & 0 & \ddots & u^{2}\left(y_{N-1}\right) & 0\\ 0 & 0 & 0 & 0 & u^{2}\left(y_{N}\right) \end{array}\right)\right),$$

and the associated part of the likelihood writes

(39) $$l\left(y|X^{*},\theta ,X\right)\propto \frac{1}{\sqrt{\det\Sigma_{y}}}\exp\left(-\frac{1}{2}{\left(y-\mu \right)}^{T}\Sigma_{y}^{-1}\left(y-\mu \right)\right),$$

where μ is defined in (33).

In this paper, we assume that the covariance matrices $\Sigma_{x}=\mathrm{diag}\left(u^{2}\left(x_{1}\right),\ldots ,u^{2}\left(x_{n}\right)\right)$ and $\Sigma_{y}=\mathrm{diag}\left(u^{2}\left(y_{1}\right),\ldots ,u^{2}\left(y_{n}\right)\right)$ are diagonal (meaning no covariance).

#### 2.5.4. Prior Distribution

The joint prior distribution $\pi \left(X^{*},\theta ,X\right)$ of all quantities involved can be expressed as the product

(40) $$\pi \left(X^{*},\theta ,X\right)=\pi \left(\theta \right)\pi \left(X^{*}\right)\pi \left(X\right),$$

where π(θ)=π(a)π(b)π(c) expresses prior information on the parameters of the calibration curve *a*, *b*, *c*, $\pi \left(X^{*}\right)$ expresses prior belief on the sought thermal conductivity $X^{*}$ and $\pi \left(X\right)$ represents the joint prior belief on the thermal conductivity measurements results stored in the vector *X*. If all measurements are assumed to be independent from each other, then $\pi \left(X\right)$ can be factorised as the product of the individual densities of probability $\pi (X_{i})$.

A variety of distributions can be used to represent prior beliefs, ranging from poorly informative to informative [45]. In this study (see Section 3.3), we choose to use non-informative Jeffrey’s priors for π(X) namely $\pi (X_{i})\propto 1$, and mildly informative priors for π(a), π(b), π(c), and $\pi (X^{*})$ as Gaussian distributions with large variance.

#### 2.5.5. Computing Posterior Distributions

Since analytical formulas for (34) and (35) are usually intractable, it is widely accepted to tackle the estimation of quantities of interest for these distributions (mean, standard deviation, coverage interval) using Markov Chain Monte Carlo (MCMC) simulations [46], from which estimations of the posterior distributions using histograms or kernel-based density estimates can also be obtained.

MCMC sampling is performed in software such as R or Python (among others), e.g., using RStan [47] as in this paper and requires statistical expertise for the tuning of the algorithms and the analysis of the results (correlation, convergence of Markov chains, etc.). A review of convergence diagnosis tools can be found in [48]. In this paper, we concentrate on the effective sample size and the Gelman-Rubin diagnostic (Rhat). In brief, the effective sample size gives the number of independent samples equivalent to a set of correlated Markov chain samples, and the output of the Gelman-Rubin diagnostic is the so-called potential scale reduction factor, which should be close to 1 and is computed from at least two chains running with over-dispersed starting points w.r.t. the posterior distribution. The great advantage of MCMC is that at each iteration, a sample from the joint posterior distribution is produced, while the collection of samples for each parameter is distributed according to its marginal posterior distribution.

## 3. Results

In this section, we implement the calibration methodology to obtain traceable estimates of the thermal conductivity using the SThM measurements and a Bayesian inversion procedure described in Section 2. We highlight that the calibration procedure is necessarily developed on bulk materials at macroscale, for which traceable thermal conductivity measurements can be obtained, and that the traceability at micrometric and nanometric scales is ensured by the SThM technique, provided that heat transfer regimes are the same between calibration and measurement, as will be discussed in Section 4.3. In Section 3.1, we display the measurement results obtained with the SThM on the calibration bulk materials presented in Table 2 according to the methodology developed in Section 2.2. In Section 3.2, we discuss the effect of the reproducibility conditions (landing and withdrawal conditions, heterogeneity of the sample) on the resulting SThM uncertainty. In Section 3.3, we illustrate the Bayesian methodology for both the identification of the parameters of the calibration curve and the prediction of traceable thermal conductivity. The approach is applied to the estimation of three thermal conductivities in the range [0.1–10] $W\;m^{-1}\;K^{-1}$.

### 3.1. Experimental Measurements on Calibration Materials

Measurements have been performed on the twelve calibration samples presented in Table 2. As described in Section 2.2, a run of 5 measurements is performed on the $SiO_{2}f$ reference sample before each run of 5 measurements on the studied calibration sample.

#### Measurements of Input Quantities for Individual Measured Quantity and Their Associated PDFs

All input quantities ($BB_{V},U,BB_{k},\ldots$) have been measured as described in Section 2.4.2, and each measured quantity $y_{m,i}$ and the associated Probability Distributions Functions (PDFs) have been determined following the method described in Section 2.4.3. Table 3 summarises the PDFs assigned to the input quantities obtained for the $y_{m,1}$ measurement on the PMMA sample as an example.

**Table 3:** Summary of PDFs assigned to measured quantity value $y_{m,1}$ for the PMMA sample.

Input Quantity	Unit	Probability Distribution	Mean Value	Standard Deviation	Lower Bound	Upper Bound
$U_{true}$	V	Gaussian	−0.004	$1.25\times 10^{-6}$	−	−
$U_{oc,q}^{s}$	V	Rectangular	−	−	$-5\times 10^{-7}$	$5\times 10^{-7}$
$U_{oc,q}^{ref}$	V	Rectangular	−	−	$-5\times 10^{-7}$	$5\times 10^{-7}$
$U_{ic,q}^{s}$	V	Rectangular	−	−	$-5\times 10^{-7}$	$5\times 10^{-7}$
$U_{ic,q}^{ref}$	V	Rectangular	−	−	$-5\times 10^{-7}$	$5\times 10^{-7}$
$U_{oc,R}^{s}$	V	Gaussian	0.37547698	$4.57\times 10^{-6}$	−	−
$U_{oc,R}^{ref}$	V	Gaussian	0.37546387	$4.08\times 10^{-6}$	−	−
$U_{ic,R}^{s}$	V	Gaussian	0.37529628	$4.13\times 10^{-6}$	−	−
$U_{ic,R}^{ref}$	V	Gaussian	0.37520403	$4.03\times 10^{-6}$	−	−
$BB_{v,true}$	V	Gaussian	−0.04	$1.25\times 10^{-6}$	−	−
$BB_{v,oc,q}^{s}$	V	Rectangular	−	−	$-5\times 10^{-7}$	$5\times 10^{-7}$
$BB_{v,oc,q}^{ref}$	V	Rectangular	−	−	$-5\times 10^{-7}$	$5\times 10^{-7}$
$BB_{v,ic,q}^{s}$	V	Rectangular	−	−	$-5\times 10^{-7}$	$5\times 10^{-7}$
$BB_{v,ic,q}^{ref}$	V	Rectangular	−	−	$-5\times 10^{-7}$	$5\times 10^{-7}$
$BB_{v,oc,R}^{s}$	V	Gaussian	$9.5697\times 10^{-3}$	$5.66\times 10^{-5}$	−	−
$BB_{v,oc,R}^{ref}$	V	Gaussian	$8.2624\times 10^{-3}$	$10.24\times 10^{-5}$	−	−
$BB_{v,ic,R}^{s}$	V	Gaussian	$-6.2327\times 10^{-3}$	$5.07\times 10^{-5}$	−	−
$BB_{v,ic,R}^{ref}$	V	Gaussian	$-14.4252\times 10^{-3}$	$3.51\times 10^{-5}$	−	−
$BB_{k}$	a. u.	Rectangular	−	−	124.5	125.5
$BB_{k,min}$	a. u.	Fixed	0.5	−	−	−
$BB_{k,max}$	a. u.	Fixed	1003	−	−	−
$R_{1}$	Ω	Rectangular	−	−	999	1001
$R_{2}$	Ω	Rectangular	−	−	999	1001
$R_{f}$	Ω	Gaussian	399.830	0.001	−	−
$R_{v,max}$	Ω	Gaussian	198.119	0.001	−	−
$R_{v,min}$	Ω	Gaussian	0.0938	0.001	−	−
$R_{1k}$	Ω	Rectangular	−	−	999	1001
$R_{1k}^{\prime }$	Ω	Rectangular	−	−	999	1001
$R_{10k}$	Ω	Rectangular	−	−	9999	10,001
$R_{10k}^{\prime }$	Ω	Rectangular	−	−	9999	10,001
$R_{1k}^{\prime \prime }$	Ω	Rectangular	−	−	999	1001

**Figure 7:**
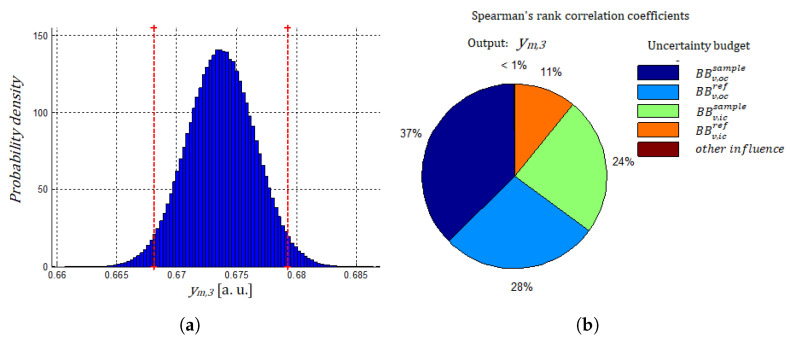
Analyses of propagation of distributions on $y_{m,3}$ measured quantity value: (**a**) Probability density distribution (PDF) for the $y_{m,3}$ value (**b**) Spearman’s rank correlation coefficients.

Based on these input quantities, we calculated the corresponding $y_{m}$ measured quantity value and determined the associated uncertainty by propagation of distributions as described in Section 2.4.3. As an example, the histogram for one value of $y_{m,i}$ is represented in Figure 7. It can be observed that the output quantity can be adequately described with a Gaussian behaviour. The Spearman study shows that the highest contribution to the variance on the individual measured quantity $y_{m}$ comes from the $BB_{v}$ measurements in “out of contact” conditions: $BB_{v,wc}^{sample}$ and $BB_{v,wc}^{ref}$ contribute to 65% of instrumental variance on $y_{m}$ measurement. This highlights the need to properly manage the environmental conditions in order to maintain constant heat transfers between the probe and its surroundings during measurement.

### 3.2. Study of Influencing Factors Regarding Repeatability and Reproducibility Conditions of Measurement

In order to estimate the measurement precision, we performed replicate measurements on each sample under repeatability and reproducibility conditions. Usually, the measurement precision is expressed numerically as the standard deviation of the set of measurements performed under specified conditions. In the process of measuring the same sample, 5 stages of landing/withdrawal are performed, which yields 5 different measurements in each configuration (in contact/out of contact). It should be noted that, in order to take account of the potential heterogeneity of the sample, measurements are performed at three different locations on the sample. In addition, the potential influence of the landing or withdrawal of the probe has also been studied. As a result, the effects of the supposedly heterogeneity of the surface of the sample and the influence of probe movement are taken into account in the dispersion of the measurements.

As a result, we obtain ten measured values $y_{m,i}$ for each calibration sample with associated uncertainty. An example of ten measured values obtained for the PMMA sample is given in Table 4. From these results, we discuss in the following Section 3.2 the repeatability and reproducibility conditions, effects, and uncertainty associated with each *Y*.

#### 3.2.1. Evaluation of Measurement Precision under Repeatability Conditions

**Figure 8:**
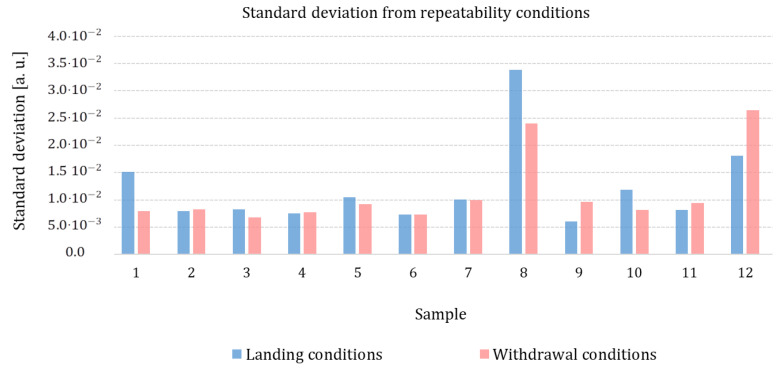
Graphic representation of the standard deviation computed from a set of three measurements performed in repeatability condition for two types of measurement (landing and withdrawal) and for all the twelve calibration samples).

For each calibration sample, we compute the standard deviation obtained with the repeatability condition for two sets of measurements: the landing condition measurement with the set composed of $y_{m,1}$, $y_{m,3}$, and $y_{m,5}$ identified measurements, and the withdrawal condition measurement with the set composed of $y_{m,2}$, $y_{m,4}$, and $y_{m,6}$ identified measurements $y_{m,i}$ as described in Table 1. Results are presented in Figure 8.

No specific trend is identified from Figure 8. Two materials ($Al_{2}O_{3}$ and Zn) show higher standard deviation values in the repeatability condition (more than $1.5\;10^{-2}$ [a.u.]) than the other materials. This is probably due to the influence of roughness. Indeed, these two materials present high roughness (respectively, 7.52 nm for $Al_{2}O_{3}$ and 8.14 nm for Zn, see Table 2). Guen [27] has shown that surface roughness alters the mechanical contact between the tip and the surface sample, reducing the apparent contact radius. When the roughness of the sample increases, heat transfer through mechanical contact decreases by 30%. As the signal decreases, the signal-to-noise ratio also decreases, and the dispersion of the measurements, evaluated by the standard deviation, increases with the roughness of the sample.

Nevertheless, it should be noted that the value of the standard deviation computed for the two sets of measurements (landing and withdrawal) is high compared with the instrumental uncertainty contribution. The values reported in Table 5 show that the relative uncertainty coming from the repeatability condition is at least double the relative uncertainty from instrumentation. This excessive dispersion of measurements suggests that the repeatability conditions are not fully respected. An influencing factor, not taken into account in this study, changes from one measurement to the next. This source of uncertainty will be discussed in Section 3.2.4.

#### 3.2.2. Evaluation of Measurement Precision under Reproducibility Conditions: Study of Landing and Withdrawal Configurations

**Figure 9:**
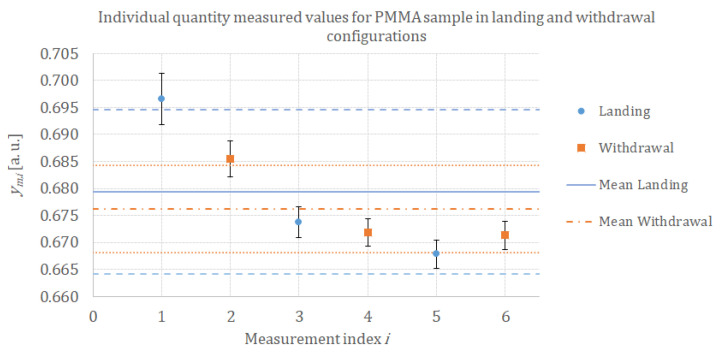
Graphic representation of the three quantity measured values of $y_{m,i,landing}$ (i=1;3 and 5), measured at the same location, computed in landing configuration and the three quantity measured values of $y_{m,i,withdrawal}$ (i=2;4 and 6) computed in withdrawal configuration. Each data is indicated with its associated absolute uncertainty (coefficient k=1) represented by black error bars. The blue circles correspond to landing measurement points and the orange square to withdrawal measurement points. The blue solid line represents the mean value for the landing configuration with its associated standard deviation (blue dashed lines) and the orange dash-dotted line represents the mean value for the withdrawal configuration with its associated standard deviation (orange dotted lines).

For each measurement, we compute the variation of resistance in two ways, as described in Section 2.2.3: landing conditions and withdrawal conditions. By this way, we check the potential influence of landing and withdrawal conditions on our measurements due to changes in heat transfers between the tip and its surroundings after contact with the sample (pollution or residual water film on the tip, variation of the surrounding temperature). We computed the mean value ${\bar{y}}_{m,landing}$ for the landing configuration with its associated expanded uncertainty and the mean value ${\bar{y}}_{m,withdrawal}$ for the withdrawal configuration with its associated expanded uncertainty. Results for the PMMA sample are presented in Figure 9. We see that there is no significant difference between the landing and withdrawal configurations for individual measurements. The mean values for ${\bar{y}}_{m,landing}$ measured quantity value in landing configuration and ${\bar{y}}_{m,withdrawal}$ in withdrawal configuration are comparable. The difference is in the same order of magnitude as the standard deviation, which means that there is no significant difference between landing and withdrawal configurations compared with the standard deviation computed from mean values.

**Figure 10:**
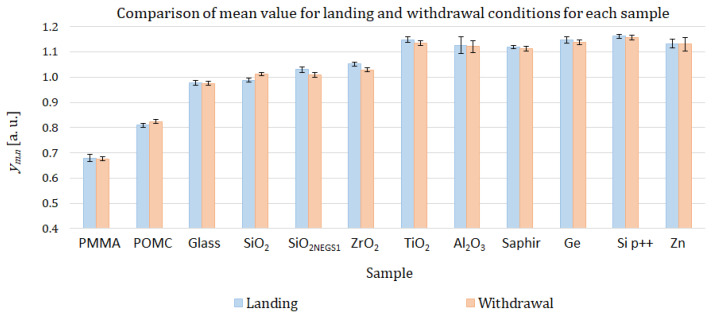
Comparison of mean values and the standard deviation (black error bars) obtained in landing condition (blue rectangles) and withdrawal condition (orange rectangles) for all calibration samples.

The same analysis has been performed for all calibration samples in order to check if there is a significant trend in mean values or standard deviation between the landing configuration and the withdrawal configuration. The data are gathered in Figure 10. No significant trend has been observed either for the mean values or for the standard deviation values.

#### 3.2.3. Evaluation of Measurement Precision under Reproducibility Conditions: Study of Heterogeneity of The Sample

**Figure 11:**
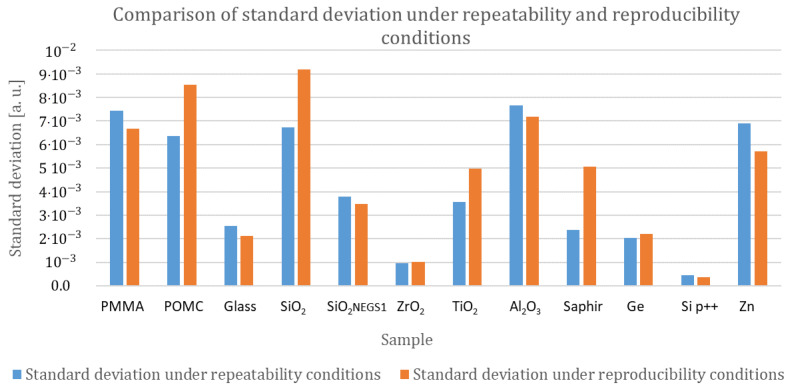
Comparison of standard deviation obtained in repeatability conditions (same location) and reproducibility conditions (different locations).

Another influence factor that can induce an additional contribution to the uncertainty is the heterogeneity of the sample, which can be due to variations in roughness from one location to another, non-uniformity (variation of structure, size grain), oxide film, or pollution. In order to identify if the heterogeneity of our samples has a significant impact on our measurement spread, for each sample, we compare in Figure 11 the standard deviation obtained for the three measurements performed at the same location and standard deviation obtained for the measurements performed at three different locations.

No significant trend indicates that sets of points measured under reproducibility conditions have a higher contribution to the standard deviation than sets of points measured under repeatability conditions. To conclude, with our measurement protocol, no significant contribution to the standard deviation has been established either from the landing or withdrawal configuration or from the heterogeneity of the sample. That means other sources of uncertainty not clearly evaluated of the purpose of the previous experiments have to be considered.

#### 3.2.4. Combination of Measurements in Repeatability and Reproducibility Conditions

**Figure 12:**
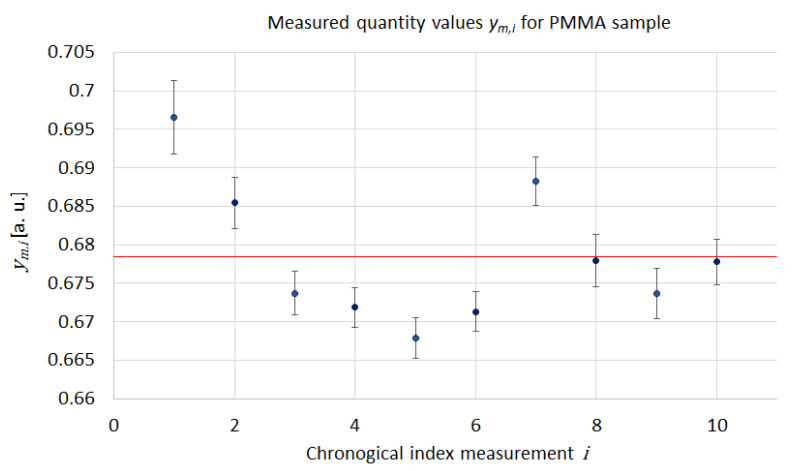
Ten measured quantity values for PMMA sample.

For each studied sample, we obtained ten quantity-measured values $Y_{m,i}$ from the repeatability and reproducibility conditions. As discussed in previous sections, the variations in repeated observations of the measurand under apparently identical conditions in highlight underestimated the uncertainty associated with the measured quantity values. As a result, we computed all the ten values measured for each sample and determined the associated uncertainty by performing Bayesian consensus estimation of the mean value of each $Y_{m,i}$ as described in Section 2.4.4. In this way, we integrate all influencing factors, even non-identified uncertainty sources, as the variation of the force applied by the cantilever between each measurement or random variation of environmental conditions. As we perform landings without the laser, we have no feedback of the deflection of the cantilever, which means no fine control of the applied force. This can induce a strong variation in the interface thermal resistance between the tip and the surface of the sample and a strong variation in the thermal contact area. This could be the main influencing factor regarding our measurement protocol. Figure 12 presents the ten quantity measured values $Y_{m,i}$ obtained for the PMMA sample with their associated uncertainty as well as the mean value identified as the intermediate measurand *y* with its associated uncertainty.

The mean values and their associated uncertainty have been computed by performing Bayesian consensus estimation for each of the twelve calibration materials. Table 6 presents results, including intermediate measurand *y*, absolute uncertainty, and relative uncertainty.

As a result, our measurement protocol enables us to reach a relative standard uncertainty of 1.0% at most. The highest values of uncertainty (≥0.7%) are obtained for materials with the highest roughness (Zn, Alumina, and POM-C). Regarding $ZrO_{2}$, even if the roughness value of its surface is quite low (<0.5nm), the relative standard uncertainty associated with the determination of $Y_{ZrO_{2}}$ is estimated to be 0.7%. A study of rough data shows that there was a variation in the thermal drift (discussed in Section 3.1 and illustrated on Figure 5) between the measurements performed on the reference $SiO_{2}$ sample and those performed on the $ZrO_{2}$ sample that increased the contribution of reproducibility to the uncertainty value. That confirms that special attention is required to the stability of environmental conditions during measurements in order to avoid increasing uncertainty.

### 3.3. Bayesian Identification of the Parameters

The Bayesian analysis proposed in Section 2.5 is carried out using the following poorly informative prior distributions for the parameters of the calibration curve: π(a)∼N(1,10), π(b)∼N(1,10), and π(c)∼N(1,10).

**Figure 13:**
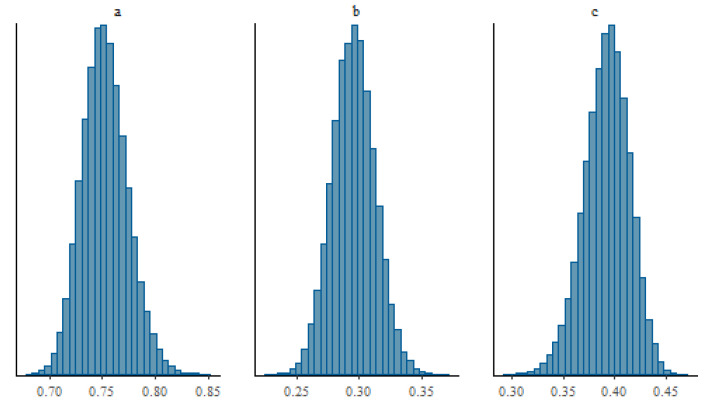
Posterior distributions of the parameters *a*, *b*, *c* of the calibration curve.

**Figure 14:**
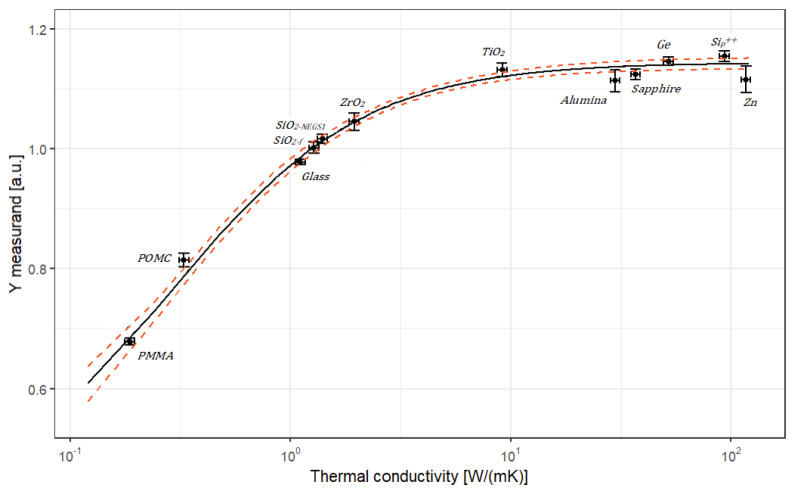
Calibration curve (in black) obtained from the experimental data from Table 6 analysed with the Bayesian approach. Points are represented with their associated expanded uncertainty (k=2) for both axes. Red dashed lines represent the 95% coverage intervals associated with the (estimated) calibration curve for each conductivity.

The posterior distributions of the parameters *a*, *b*, and *c* of the calibration curve are presented in Figure 13. The corresponding calibration curve is illustrated in Figure 14 with its 95% coverage intervals.

The calibration curve provided in Figure 14 suggests that the SThM technique is a promising technique for the determination of traceable thermal conductivities lower than 10 $W\;m^{-1}\;K^{-1}$ in the best case. Due to the shape of the calibration curve, the sensitivity of the technique highly decreases for thermal conductivity higher than 10 $W\;m^{-1}\;K^{-1}$, which means that uncertainties on measurement for high thermally conductive materials should be significant values that will be discussed in Section 3.4.

### 3.4. Predictions and Associated Uncertainty Using the Calibration Curve

In this section, we study predictions of the thermal conductivity and their associated uncertainties $X^{*}$ using the calibration curve for arbitrary intermediate measurand $Y^{*}$. In order to cover the range [0.1,10] $W\;m^{-1}\;K^{-1}$ of thermal conductivity, the following three artificial $y^{*}$ measurements are chosen to illustrate the prediction using the Bayesian methodology: $y_{1}^{*}=0.7$, $y_{2}^{*}=1.11$, and $y_{3}^{*}=1.12$. In this case, the vectors of SThM measurements $Y^{*}$ and predictions $X^{*}$ of the thermal conductivity write $Y^{*}=\left(Y_{1}^{*},Y_{2}^{*},Y_{3}^{*}\right)$ and $X^{*}=\left(X_{1}^{*},X_{2}^{*},X_{3}^{*}\right)$ respectively (see Section 2.5.1).

The following poorly informative prior distributions are chosen for the predictions $\pi \left(X_{1}^{*}\right)∼N\left(0.5,10\right)$, $\pi \left(X_{2}^{*}\right)∼N\left(5,100\right)$, $\pi \left(X_{3}^{*}\right)∼N\left(10,100\right)$ and non informative Jeffrey’s prior distributions are chosen for the $X_{i}$: $\pi (X_{i})\propto 1$ for i∈1,…,N.

In this study, we consider two uncertainty levels for $y^{*}$, namely $u(y^{*})=0.005$, which corresponds to the median of observed uncertainties obtained with the SThM on the bulk materials in this study, and $u(y^{*})=0.002$, which corresponds to the lowest observed uncertainty. Since all parameters are jointly updated (see Section 2.5.2), we show that the uncertainty level of $y^{*}$ used for prediction has an effect on the estimations of all the parameters, in particular on those of the calibration curve, which is a desirable feature of the Bayesian inference. Summaries of the posterior distributions of all parameters $\left(a,b,c,X_{1},\ldots ,X_{N},X_{1}^{*},X_{2}^{*},X_{3}^{*},Y_{1},\ldots ,Y_{N},Y_{1}^{*},Y_{2}^{*},Y_{3}^{*}\right)$ with N=12 are displayed in Appendix A Table A1 and Table A2 for $u(y^{*})=0.005$ and $u(y^{*})=0.002$, respectively.

**Figure 15:**
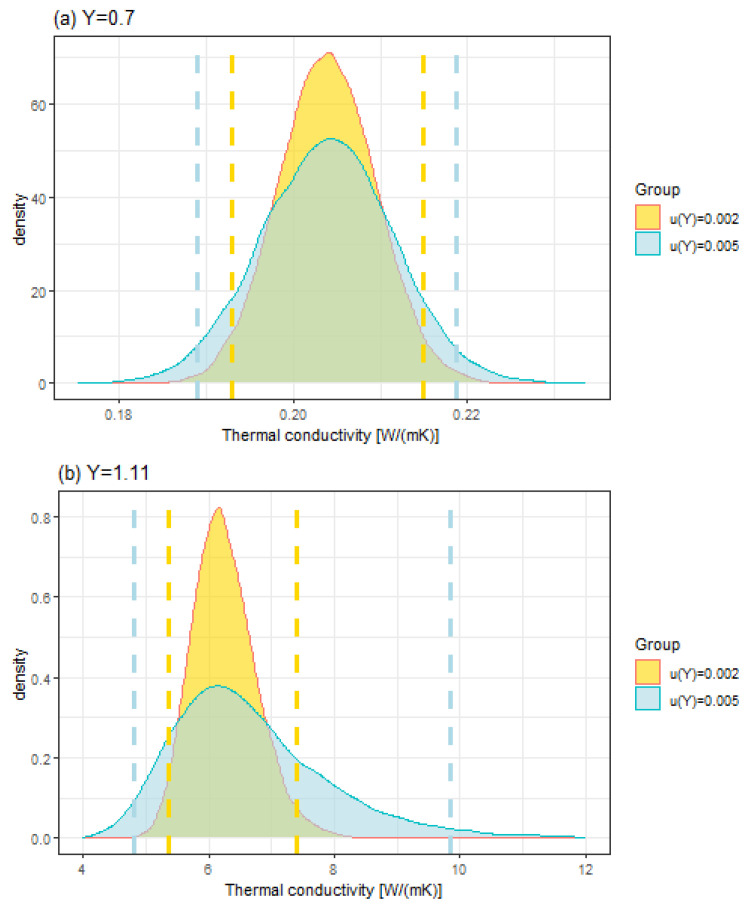

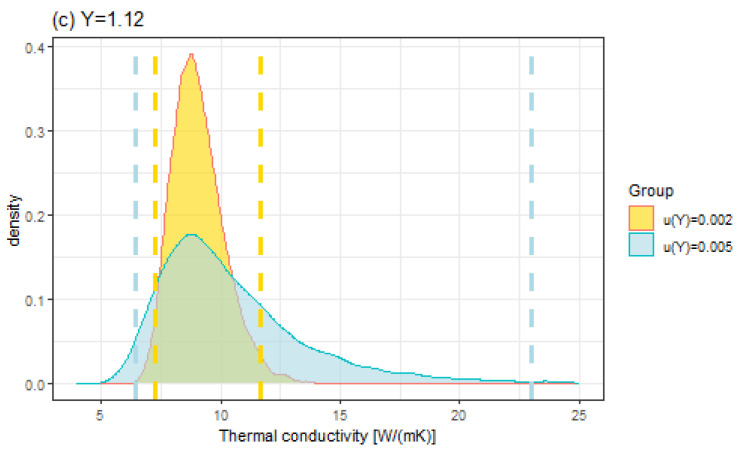
Posterior distributions of the predictions and their associated 95% coverage interval obtained for (**a**) $Y=y_{1}^{*}$, (**b**) $Y=y_{2}^{*}$ and (**c**) $Y=y_{3}^{*}$ for the two uncertainty levels $u(y^{*})=0.002$ and $u(y^{*})=0.005$.

The effect of a lower uncertainty $u(y^{*})=0.002$ on the predictions from the calibration curve is displayed in Section 4.1. The posterior distributions of the predictions and their associated 95% coverage interval obtained for $y_{1}^{*}=0.7$, $y_{2}^{*}=1.11$, and $y_{3}^{*}=1.12$ are presented in Figure 15 for the two uncertainty levels $u(y^{*})=0.002$ and $u(y^{*})=0.005$. It should be noted that the posterior distributions of the predictions are not symmetric PDFs, in particular for the highest conductivity. The predicted values are given with their associated 95% coverage interval. The highest density value for the predicted value is not located at the centre of the coverage interval. Therefore, it is not correct to attribute a standard uncertainty (absolute or relative) to the predicted value. Only the coverage interval provides a rigorous assessment of the uncertainty.

## 4. Discussions

### 4.1. Sensitivity of the Measurement Method

The calibration curve provided in Figure 14 suggests that the SThM technique is a promising technique for the determination of thermal conductivities lower than 10 $W\;m^{-1}\;K^{-1}$. In this section, we propose to illustrate this finding with three different values of the *Y* measurements. Table 7 provides the results of the prediction obtained from simulation techniques based on MCMC for the three chosen values of the *Y* intermediate measurand. Two levels of standard uncertainty associated with *Y* measurements have been tested: 0.002 and 0.005.

As expected, the uncertainty associated with the thermal conductivity increases as the *Y* intermediate measurand approaches the top of the rising part of the calibration curve in Figure 14. It appears that, currently, for values of *Y* higher than 1.2, which corresponds to a thermal conductivity of $10\;W\;m^{-1}\;K^{-1}$, the model does not enable the prediction of a thermal conductivity value with a sufficiently low uncertainty. The second important result presented in Table 7 is that the control of the uncertainty associated with the intermediate measurand *Y* is crucial. The simulation performed for a *Y* value of 1.2 with an associated uncertainty of 0.005 instead of 0.002 shows that it becomes impossible to determine the thermal conductivity with a sufficiently low uncertainty.

### 4.2. Improvement of Measurement Precision

As discussed in Section 3.2, the main contribution to the standard uncertainty is the dispersion of values in readability and reproducibility conditions. This statistical spread between measurements could be explained by heterogeneities at the surface of the sample (the contribution of reproducibility error to the variance seems to decrease a little bit when the roughness of materials decreases, see Table 2 and Table 6). Nevertheless, given the small decrease in the contribution of the reproducibility (at best, one point), it seems that the spread is also impacted by changes in the thermal probe-sample contact between each measurement (thermal interface resistance, thermal contact area, etc.). Since we follow the “dark mode” protocol to avoid the overheating induced by the laser, we do not manage accurately the applied tip force from one measurement to the next. Variations in applied tip force between measurements could induce changes in thermal interface resistance and thermal contact area. The next step of this work is to improve the reproducibility of our measurements by improving the management of the applied tip force.

### 4.3. Application to Nanomaterials

Estimating the thermal conductivity of an unknown material from the calibration curve requires that the heat transfer conditions are the same during the calibration and measurement. As the calibration curve has been established with bulk materials, the heat flows are mainly diffusive, which means that thermal conductivity can be directly identified only for nanostructures or materials whose dimensions are consistent with the Fourier regime with local thermodynamic equilibrium [49]. In the probe-sample-environment system, at least three dimensions have to be checked: the smallest dimension of the sample $d_{min}$ (such as the diameter of the nanowire or the thickness of the membrane), the mean-free path Λ of the studied material, and the effective solid-solid contact radius $b_{eff}$ between the probe and the sample.

For systems where at least one of the dimensions $d_{min}$ or $b_{eff}$ is lower than the mean-free path Λ, heat transport is completely different from that experienced in macroscopic systems. In that case, identification of the thermal conductance requires the development of specific model describing all heat transfers from the probe to the sample (including ballistic effects). To build the model, it is necessary to have a complete knowledge of the geometry of the probe (depending on the probe type, probe generation, or wear of the probe) [49,50], information about the thermal and electrical properties of the materials of the probe, and a perfect knowledge of the contact between the tip and the sample (surface roughness, water meniscus, boundary resistance, …) [15]. Unfortunately, it is really challenging to quantify all quantities involved in the measurement of the thermal conductivity of material and in the heat transfer model [50]. Indeed, measuring the thermal or electrical properties of the material constituents of the probe is tricky due to their small sizes. In addition, some parameters of the model and heat transfer mechanisms at a lower scale than the mean-free path are still the subject of fundamental research.

## 5. Conclusions

This work provides the first complete uncertainty assessment of thermal conductivity measurements by the SThM technique based on a calibration curve established with bulk calibration materials. This study shows that following the proposed protocols, it is possible to perform quantitative and traceable thermal conductivity measurements for materials with low thermal conductivity (under 10 $W\;m^{-1}\;K^{-1}$) with the SThM technique under specific conditions. Traceability is ensured by using calibration materials whose thermal conductivity measurements are themselves traceable. As traceability is established at the macroscale on bulk calibration materials, the measurement conditions must be the same between calibration and measurements (i.e., diffusive heat transfer regime). By ensuring strictly steady-state environmental conditions, minimising the roughness of studied material (ideally less than $R_{a}<1\;\mathrm{nm}$), and minimising the dispersion of measurements to limit uncertainty on the measured quantity value to 0.2%, it is possible to reach an uncertainty value of less than 10% for the identified thermal conductivity value. For thermal conductivity greater than 10 $W\;m^{-1}\;K^{-1}$, the current uncertainty values are too high from a metrological point of view to justify traceable measurement using the protocol developed for this study.

Regarding nanostructured materials, traceable measurement could be performed using the calibration and measurement protocols described in this paper when the heat transfer regimes stay the same between calibration and measurement, that is, in the diffusive regime. For a probe-sample system with one dimension lower than the mean-free path of the studied material, a dedicated model has to be developed, as highlighted in ref. [50].

## Figures and Tables

**Table 1:** Identification of the ten measured values $y_{m,i}$ from various conditions of measurement indexed from i=1 to i=10 relative to the chronological acquisition, odd *i* indexes relative to landing measurement condition, even *i* indexes relative to withdrawal measurement condition.

Locations	Landing Condition	Withdrawal Condition
location n°1	$y_{m,1}$	$y_{m,2}$
	$y_{m,3}$	$y_{m,4}$
	$y_{m,5}$	$y_{m,6}$
location n°2	$y_{m,7}$	$y_{m,8}$
location n°3	$y_{m,9}$	$y_{m,10}$

**Table 2:** Thermal conductivity *k* (relative expanded uncertainty estimated to 5%) and roughness Ra of calibrated materials measured at 23 °C. The sample thickness is identified as: 1 *, 2 ** and 5 *** mm.

Sample	Structure	Provider	*k* [Wm${}^{-1}$K${}^{-1}$]	Ra [nm]
PMMA ***	Polymer	Goodfellow	0.187	5.04
POM−C ***	Polymer	Radiospare	0.329	11.7
Borosilicateglass **	Amorphous	Neyco	1.11	<0.5
$SiO_{2}f$ **	Amorphous	Neyco	1.28	0.56
$SiO_{2-NEGS1}$ **	Amorphous	Neyco	1.40	<1
$ZrO_{2}$ **	Single crystal	Neyco	1.95	<0.5
$TiO_{2}$ **	Single crystal	Neyco	9.15	<0.5
$Al_{2}O_{3}p$ **	Poly crystal	Neyco	29.8	7.52
Sapphire *	Single crystal	Crystal GmbH	36.9	<0.5
Germanium **	Single crystal	Crystal GmbH	52.0	<0.5
$Sip^{++}$ **	Semiconductor	Goodfellow	93.4	0.75
Zinc **	Metal	Neyco	117	8.14

**Table 4:** Summary of the ten values obtain on the PMMA sample: the measured quantity value $y_{m,i}$, the standard uncertainty (absolute and relative) and the 95% coverage interval are given.

Identification Measurement	$y_{m,i}$ Value [a. u.]	Standard Uncertainty		95% Coverage Interval	
		Abs	Rel. (%)	[a. u.]	[a. u.]
$y_{m,1}$	0.6966	0.0047	0.68	0.6873	0.7058
$y_{m,2}$	0.6855	0.0033	0.48	0.6789	0.6919
$y_{m,3}$	0.6737	0.0028	0.42	0.6681	0.6792
$y_{m,4}$	0.6719	0.0025	0.38	0.6668	0.6768
$y_{m,5}$	0.6679	0.0026	0.39	0.6628	0.6731
$y_{m,6}$	0.6713	0.0026	0.38	0.6663	0.6764
$y_{m,7}$	0.6883	0.0032	0.46	0.6821	0.6944
$y_{m,8}$	0.6780	0.0034	0.50	0.6714	0.6848
$y_{m,9}$	0.6737	0.0033	0.49	0.6673	0.6801
$y_{m,10}$	0.6778	0.0030	0.44	0.6719	0.6836

**Table 5:** Comparison of instrumental standard uncertainty value associated to each measured quantity value to the standard deviation value computed for set of replicated measurements for each materials. The highest instrumental standard uncertainties (absolute and relative) and the computed standard deviation (absolute and relative) are given.

Material Sample	Measurement Condition	Max. Standard Uncertainty		Standard Deviation	
		Abs	Rel. (%)	Abs	Rel. (%)
PMMA	landing	0.0047	0.68	0.0152	2.23
	withdrawal	0.0034	0.50	0.0080	1.19
POM−C	landing	0.0031	0.38	0.0080	0.98
	withdrawal	0.0034	0.41	0.0083	1.01
Borosilicateglass	landing	0.0045	0.46	0.0083	0.85
	withdrawal	0.0045	0.47	0.0068	0.70
$SiO_{2}f$	landing	0.0049	0.49	0.0075	0.76
	withdrawal	0.0043	0.43	0.0078	0.77
$SiO_{2}-NEGS1$	landing	0.0051	0.50	0.0105	1.02
	withdrawal	0.0044	0.44	0.0074	0.70
$ZrO_{2}$	landing	0.0052	0.49	0.074	0.70
	withdrawal	0.0047	0.46	0.0074	0.72
$TiO_{2}$	landing	0.0056	0.49	0.0101	0.88
	withdrawal	0.0057	0.50	0.0100	0.88
Alumina	landing	0.0051	0.45	0.0338	3.00
	withdrawal	0.0050	0.45	0.024	2.14
Sapphire	landing	0.0038	0.34	0.0061	0.54
	withdrawal	0.0039	0.35	0.0096	0.86
Germanium	landing	0.0045	0.39	0.0118	1.03
	withdrawal	0.0051	0.45	0.0082	0.72
$Sip^{++}$	landing	0.0042	0.36	0.0082	0.71
	withdrawal	0.0041	0.35	0.0094	0.81
Zinc	landing	0.0055	0.48	0.0181	1.59
	withdrawal	0.0055	0.49	0.0264	2.34

**Table 6:** Summary of the experimental measurements on calibration samples: the intermediate measurand mean value *Y*, the standard uncertainty u(Y) (absolute and relative).

Sample	Thermal Conductivity	*Y* Intermediate Measurand	u(Y) Standard Uncertainty	
	(Wm${}^{-1}$K${}^{-1}$)	Mean Value (a.u.)	Abs	Rel.(%)
PMMA	0.187	0.6780	0.0029	0.4
POM−C	0.329	0.8145	0.0055	0.7
Borosilicateglass	1.11	0.9780	0.0023	0.2
$SiO_{2}f$	1.28	1.0019	0.0048	0.5
$SiO_{2}-NEGS1$	1.40	1.0173	0.0038	0.4
$ZrO_{2}$	1.95	1.0457	0.0072	0.7
$TiO_{2}$	9.15	1.1316	0.0057	0.5
Alumina	29.8	1.1140	0.0091	0.8
Sapphire	36.9	1.1241	0.0045	0.4
Germanium	52.0	1.1460	0.0035	0.3
$Sip^{++}$	93.4	1.1548	0.0044	0.4
Zinc	117	1.1158	0.0111	1.0

**Table 7:** Prediction of the thermal conductivity for different values of the direct measurement. The indications of the standard uncertainty (absolute and relative) are given only for the order of magnitude, only the coverage interval give the rigorous estimation of the uncertainty level as discussed in previous section (Section 3.4).

$y_{0}$	$u\left(y_{0}\right)$	$k_{0}$ **Wm${}^{-1}$K${}^{-1}$**	$u\left(k_{0}\right)$ **Wm${}^{-1}$K${}^{-1}$**	$u\left(k_{0}\right)$ **(**%**)**	95% Coverage Interval **Wm${}^{-1}$K${}^{-1}$**
0.7	0.005	0.20407	0.00753	3.7	$\left[0.18923;0.21897\right]$
0.7	0.002	0.20390	0.00560	2.7	$\left[0.19296;0.21493\right]$
1.1	0.005	6.69603	1.29795	19.4	$\left[4.83485;9.77990\right]$
1.1	0.002	6.25423	0.51752	8.3	$\left[5.36013;7.39395\right]$
1.2	0.005	11.01500	5.21046	47.3	$\left[6.48843;22.57146\right]$
1.2	0.002	9.07703	1.13082	12.5	$\left[7.28483;11.65243\right]$

## Data Availability

Data that support the findings of this study are available on Zenedo repository: https://doi.org/10.5281/zenodo.8188536; URL (accessed on 29 July 2023); Fleurence, Nolwenn; Douri, Sarah; Demeyer, Séverine; Allard, Alexandre and Hay, Bruno. (2023). Measurements on reference materials for SThM measurand versus thermal conductivity calibration curve (Version v1) [Data set].

## Abbreviations

### Abbreviations

The following abbreviations are used in this manuscript:

AFM	Atomic Force Microscopy
a. u.	arbitrary unit
DC	Direct current
GUM	Guide to the expression of Uncertainty in Measurement
MCM	Monte Carlo Method
MCMC	Markov Chain Monte Carlo
PDF	Probability Distribution Functions
PMMA	poly(methyl methacrylate)
POM-C	poly-oxymethylene in copolymer
SEM	Scanning Electron Microscopy
SI	International System of Units (SI for Système International)
SThM	Scanning Thermal Microscopy
TCR	Temperature Coefficient Ratio

### Nomenclature

**Measurement Result**	**Measured Quantity Value**	**Uncertainty**	**Description**
$Y_{m}$	$y_{m}$	$u(y_{m})$	individual measurand
$Y_{m,i}$	$y_{m,i}$	$u(y_{m,i})$	individual measurand indexed by environmental measurement conditions
*Y*	*y*	u(y)	mean value of measurand

## Appendix A. Bayesian Estimates

**Table A1:** Posterior point estimates of all the quantities updated in the Bayesian inference for u(Y)=0.005.

	Mean	SD	2.50%	25%	50%	75%	97.50%	n_eff	Rhat
*a*	0.75248	0.02123	0.71332	0.73791	0.75154	0.76631	0.79741	18891	1.000
*b*	0.29479	0.01695	0.26192	0.28337	0.29456	0.30605	0.32837	18534	1.000
*c*	0.39178	0.02189	0.34578	0.37749	0.39274	0.40688	0.43207	18832	1.000
$X_{1}$	116.98244	2.93226	111.14404	115.03346	116.97302	118.97162	122.66783	18263	1.000
$X_{2}$	93.51105	2.33664	89.00636	91.91874	93.49173	95.09355	98.13226	18588	1.000
$X_{3}$	52.08016	1.30062	49.51823	51.21204	52.08048	52.94909	54.64027	19055	1.000
$X_{4}$	36.81147	0.92839	34.99854	36.18567	36.81277	37.44119	38.65114	19178	1.000
$X_{5}$	29.77573	0.73586	28.33211	29.28152	29.77346	30.26941	31.23389	19163	1.000
$X_{6}$	9.19203	0.22784	8.74616	9.03959	9.18998	9.34594	9.63890	18822	1.000
$X_{7}$	1.95029	0.04671	1.85811	1.91892	1.95029	1.98190	2.04136	19503	1.000
$X_{8}$	1.41600	0.03044	1.35605	1.39574	1.41594	1.43636	1.47612	18841	1.000
$X_{9}$	1.27543	0.02837	1.21952	1.25637	1.27541	1.29451	1.33108	18796	1.000
$X_{10}$	1.06069	0.02177	1.01842	1.04592	1.06073	1.07540	1.10332	18052	1.000
$X_{11}$	0.34749	0.00670	0.33456	0.34297	0.34752	0.35199	0.36062	19347	1.000
$X_{12}$	0.18227	0.00451	0.17349	0.17918	0.18223	0.18534	0.19105	19384	1.000
$X_{1}^{*}$	0.20387	0.00760	0.18887	0.19872	0.20396	0.20896	0.21867	19061	1.000
$X_{2}^{*}$	6.69274	1.31602	4.80659	5.79299	6.46207	7.33991	9.85410	17246	1.000
$X_{3}^{*}$	11.09852	5.61346	6.44979	8.35621	9.83874	12.09348	22.99046	15898	1.000
$Y_{1}$	1.14237	0.00220	1.13801	1.14090	1.14236	1.14386	1.14668	17798	1.000
$Y_{2}$	1.14190	0.00220	1.13756	1.14043	1.14190	1.14338	1.14620	17817	1.000
$Y_{3}$	1.14003	0.00216	1.13575	1.13860	1.14004	1.14148	1.14427	17747	1.000
$Y_{4}$	1.13829	0.00212	1.13409	1.13687	1.13830	1.13971	1.14244	17864	1.000
$Y_{5}$	1.13689	0.00210	1.13272	1.13548	1.13689	1.13830	1.14100	17859	1.000
$Y_{6}$	1.12090	0.00191	1.11714	1.11962	1.12091	1.12217	1.12462	17463	1.000
$Y_{7}$	1.04558	0.00275	1.04010	1.04375	1.04559	1.04744	1.05089	19108	1.000
$Y_{8}$	1.01479	0.00265	1.00958	1.01300	1.01477	1.01658	1.01997	19042	1.000
$Y_{9}$	1.00319	0.00305	0.99713	1.00114	1.00321	1.00529	1.00906	18628	1.000
$Y_{10}$	0.98086	0.00209	0.97674	0.97949	0.98087	0.98226	0.98490	18485	1.000
$Y_{11}$	0.79942	0.00389	0.79171	0.79685	0.79940	0.80204	0.80706	18576	1.000
$Y_{12}$	0.67998	0.00289	0.67432	0.67804	0.67999	0.68191	0.68565	19186	1.000
$Y_{1}^{*}$	0.70005	0.00503	0.69018	0.69666	0.70009	0.70344	0.70986	16720	1.000
$Y_{2}^{*}$	1.11155	0.00517	1.10141	1.10807	1.11154	1.11503	1.12171	17568	1.000
$Y_{3}^{*}$	1.12256	0.00540	1.11209	1.11894	1.12240	1.12612	1.13366	19017	1.000

**Table A2:** Posterior point estimates of all the quantities updated in the Bayesian inference for u(Y)=0.002.

	Mean	SD	2.50%	25%	50%	75%	97.50%	n_eff	Rhat
*a*	0.75257	0.02113	0.71390	0.73786	0.75145	0.76632	0.79607	18663	1
*b*	0.29500	0.01698	0.26260	0.28346	0.29471	0.30641	0.32894	18710	1
*c*	0.39177	0.02181	0.34678	0.37757	0.39288	0.40693	0.43158	18633	1
$X_{1}$	116.98164	2.94698	111.28035	114.99642	116.94231	118.95543	122.83754	19253	1
$X_{2}$	93.50339	2.32200	88.98024	91.94296	93.52646	95.04895	98.07742	18317	1
$X_{3}$	52.06313	1.30199	49.49339	51.17531	52.07019	52.94611	54.60207	18996	1
$X_{4}$	36.81481	0.92593	34.98734	36.18401	36.81396	37.43419	38.62662	18505	1
$X_{5}$	29.75853	0.74063	28.29387	29.25888	29.76038	30.25958	31.20828	18635	1
$X_{6}$	9.19174	0.22802	8.74244	9.03749	9.19330	9.34511	9.63806	18499	1
$X_{7}$	1.95060	0.04698	1.85851	1.91921	1.95035	1.98212	2.04297	19210	1
$X_{8}$	1.41630	0.03024	1.35708	1.39610	1.41610	1.43617	1.47689	18904	1
$X_{9}$	1.27500	0.02860	1.21919	1.25568	1.27521	1.29422	1.33215	18075	1
$X_{10}$	1.06051	0.02194	1.01763	1.04562	1.06045	1.07525	1.10400	19529	1
$X_{11}$	0.34759	0.00669	0.33440	0.34305	0.34761	0.35208	0.36065	18154	1
$X_{12}$	0.18230	0.00448	0.17364	0.17924	0.18230	0.18531	0.19120	18516	1
$X_{1}^{*}$	0.20390	0.00560	0.19296	0.20014	0.20390	0.20767	0.21493	18498	1
$X_{2}^{*}$	6.25423	0.51752	5.36013	5.89422	6.21048	6.56798	7.39395	18765	1
$X_{3}^{*}$	9.07703	1.13082	7.28483	8.28182	8.93303	9.72300	11.65243	18740	1
$Y_{1}$	1.14245	0.00218	1.13822	1.14096	1.14245	1.14393	1.14673	18174	1
$Y_{2}$	1.14197	0.00217	1.13775	1.14049	1.14198	1.14344	1.14623	18172	1
$Y_{3}$	1.14010	0.00213	1.13597	1.13865	1.14011	1.14155	1.14428	18083	1
$Y_{4}$	1.13836	0.00210	1.13428	1.13693	1.13836	1.13979	1.14247	18160	1
$Y_{5}$	1.13696	0.00207	1.13292	1.13554	1.13697	1.13836	1.14103	18196	1
$Y_{6}$	1.12096	0.00188	1.11730	1.11969	1.12097	1.12222	1.12463	18463	1
$Y_{7}$	1.04560	0.00276	1.04017	1.04375	1.04561	1.04748	1.05101	19187	1
$Y_{8}$	1.01480	0.00267	1.00960	1.01301	1.01480	1.01659	1.02002	18771	1
$Y_{9}$	1.00313	0.00306	0.99707	1.00106	1.00317	1.00523	1.00903	18424	1
$Y_{10}$	0.98081	0.00209	0.97675	0.97939	0.98081	0.98223	0.98490	19062	1
$Y_{11}$	0.79938	0.00387	0.79179	0.79677	0.79937	0.80202	0.80693	17955	1
$Y_{12}$	0.67991	0.00290	0.67425	0.67792	0.67989	0.68183	0.68559	19199	1
$Y_{1}^{*}$	0.70004	0.00202	0.69606	0.69867	0.70002	0.70141	0.70400	19015	1
$Y_{2}^{*}$	1.11027	0.00200	1.10633	1.10894	1.11026	1.11160	1.11422	19024	1
$Y_{3}^{*}$	1.12034	0.00204	1.11636	1.11896	1.12033	1.12170	1.12432	18750	1
